# Regulatory T cell heterogeneity: who they are, where they come from, and where they go

**DOI:** 10.3389/fimmu.2026.1929923

**Published:** 2026-10-09

**Authors:** Fan Yang, Yasuhiro Ikeda, Zengli Guo, Kyle J. Bednar

**Affiliations:** 1 Cell Therapy Advanced Technologies, Oncology R&D, AstraZeneca, Gaithersburg, MD, United States; 2 Bioscience Immunology, Research and Early Development, Respiratory and Immunology, Biopharmaceuticals R&D, AstraZeneca, Gaithersburg, MD, United States

**Keywords:** adoptive cell therapy, effector Treg, immune tolerance, naïve Treg, regulatory T cells, Treg heterogeneity

## Abstract

Regulatory T cells (Tregs) are essential for enforcing immune tolerance and homeostasis, but they comprise several distinct populations that differ in developmental origin, activation state, tissue adaptation, and functional specialization. These differences shape where Tregs reside, which immune responses they suppress, and how stable their lineage remains over time. This heterogeneity matters increasingly as immunology and therapeutics move from bulk Treg measurements toward subset- and context-specific interpretations. Treg-directed strategies now range from expanding Tregs to treat autoimmune disease to inhibiting tumor Tregs to enhance tumor immunity. In this review, we use three questions (who heterogeneous Tregs are, where they come from, and where they go) to organize recent advances that explain how Tregs are generated and maintained, how they traffic and adapt across tissues, and how their states shift across physiology and diseases. We also delineate Treg heterogeneity with a particular focus on contrasts between naïve Tregs (nTregs) and effector Tregs (eTregs), including defining markers and key biological features. We aim to provide a practical framework for interpreting human Treg datasets and for selectively using distinct Treg subsets in experimental design and therapeutic development.

## 1 Introduction (who they are: definitions, identity, and core functions of Treg)

Tregs were discovered as a lineage of immunosuppressive CD25^+^CD4^+^ T cells, whose functionality and identity are defined by constitutive expression of the transcription factor FOXP3, a concept established by seminal genetic and mechanistic studies underpinning the 2025 Nobel recognition (1–3). Beyond FOXP3 level, epigenetic demethylation at conserved non-coding sequence 2 (CNS2; also termed the Treg-specific demethylated region, TSDR) within the *FOXP3* locus, and at additional Treg signature gene loci (such as *IL2RA*, *IKZF4*, and *CTLA4*) secures Treg identity, discriminating stable Treg from activated FOXP3^+^ conventional T cells (4–7). Tregs mediate immune suppression through complementary mechanisms: high CD25-mediated IL-2 consumption that limits IL-2 availability to IL-2-sensitive effector T cells (Teffs); inhibitory receptors (for example, CTLA-4, PD-1, TIGIT); inhibitory cytokines including IL-10, TGF-β, and IL-35; ecto-enzymatic conversion of extracellular ATP to anti-inflammatory adenosine via CD39/CD73; and cytotoxic modules such as granzyme B and perforin (8). In addition to immune modulation, Tregs contribute to tissue homeostasis and repair by adapting to local cues (such as damage signals) and producing effector molecules such as amphiregulin, TGF-β, JAG1, IL-10, osteopontin, and SPARC to support epithelial, mesenchymal, endothelial, and neural regeneration across organs (9–11). We and others have recently reviewed the various tissue-specific roles of Treg (12, 13).

Soon after the identification of human Tregs, circulating CD4^+^CD25^high^ cells were found to be predominantly CD45RO^+^ activated/effector Tregs (eTregs) (14), with a smaller fraction (approximately 0.2–3.3% of total CD4^+^ T cells) comprising CD45RA^+^ naïve/resting Tregs (nTregs) (15, 16). Subsequent work delineated human blood CD4^+^CD25^+^ cells into three fractions: Fraction I (Fr. I) CD45RA^+^FOXP3^int^/CD25^int^ nTreg, Fr. II CD45RA^−^FOXP3^high^/CD25^high^ eTreg, and Fr.III CD45RA^-^FOXP3^lo^/CD25^lo^, a mixed population that includes non-Tregs and a minority of Tregs (17, 18). Tregs are plastic and can adapt to different immune environments by differentiating into specialized subtypes, such as T helper 1 (Th1)-, Th2-, Th17-like Treg and follicular Treg (Tfr), to enhance context-matched immune modulation (19). A separate classification concerns developmental origin: thymus-derived Tregs (tTregs) differentiate from Treg precursors during thymic development, whereas peripherally derived Tregs (pTregs) arise from FOXP3^−^ conventional CD4^+^ T cells in peripheral tissues under tolerogenic conditions (**Table 1**). In humans, no individual marker definitively distinguishes tTregs from pTregs. Although circulating CD45RA^+^ nTregs are generally enriched for cells of thymic origin, nTreg and tTreg are not synonymous. Conversely, eTregs may include antigen-experienced tTregs and pTregs; therefore, pTreg and eTreg should not be used interchangeably. Tregs may also be classified according to the mode of generation. Naturally occurring Tregs are generated *in vivo* and include both tTregs and pTregs. In contrast, *in vitro*-induced Tregs (iTregs) are generated *ex vivo* from conventional T cells using defined stimulation and culture conditions (20) (**Table 1**). Historical terminology is potentially confusing (**Table 1**); to avoid confusion, we use nTreg and eTreg exclusively to refer to the naïve and effector Tregs in this review, while referencing the 2013 nomenclature that defines tTreg (thymus-derived), pTreg (peripherally derived), and iTreg cell (*in vitro*–induced) (20) (**Table 1**). Lineages such as Type 1 and Type 2 regulatory T cells (Tr1 and Tr2) and CD8^+^ Tregs, which do not express FOXP3 (21), are outside the scope of this review.

**Table 1:** Terminology for major subtypes of human Treg.

Category	Alternative terminology in the literature	Nomenclature recommended in 2013		Nomenclature used in this review
Activation state	Antigen-inexperienced/Resting	Naïve Treg (**nTreg**)/resting Treg (**rTreg**)	–	Naïve Treg **(nTreg)**
	Antigen-experienced/Activated	Effector Treg (**eTreg**)/activated Treg (**aTreg**)	–	Effector Treg (**eTreg**)
Developmental origin	Thymus: derived from precursor cells	thymus-derived Treg (**tTreg**)/central Treg (**cTreg**)/sometimes also called natural Treg (**nTreg**)	**tTreg**	thymus-derived Treg **(tTreg)**
	Periphery (extrathymic): derived from naïve or effector CD4 T cell	peripheral-derived Treg (**pTreg**)/sometimes also called adaptive Treg cell (**aTreg**)/sometimes also called induced Treg (**iTreg**)	**pTreg**	peripheral-derived Treg **(pTreg)**
Mode of generation	Generated physiologically *in vivo*	naturally occurring Treg (**nTreg**)	–	naturally occurring Treg (**nTreg**)
	Generated from conventional T cells *ex vivo* using defined culture conditions	*In vitro*–induced Treg (**iTreg**)	**iTreg**	*In vitro*–induced Treg **(iTreg)**

Different Treg populations differ in origin, stability, signaling/metabolic wiring, and therapeutic suitability, all of which directly affect adoptive Treg manufacturing and Treg-targeted modalities. In this review, we synthesize the differentiation and trafficking of different subsets of Treg, how major Treg states (especially nTreg and eTreg) are defined, and how this heterogeneity can be leveraged to design more precise and durable Treg-based therapies.

## 2 Where they come from: differentiation and trafficking trajectories of Treg

### 2.1 Treg development in the thymus

Thymic Treg development is primarily defined in mice and has been comprehensively reviewed in (22–24). Briefly, at the double positive (DP) and early CD4 single positive (SP) stages, Satb1 primes future Treg super-enhancers by binding closed chromatin. This priming enables subsequent chromatin opening, activation-linked histone modifications, transcription factor recruitment, and Treg-specific DNA hypomethylation at key loci such as *Foxp3* CNS regions, *Il2ra*, and *Ctla4* (23, 25). During the CD4 SP phase, TCR signal strength helps determine fate among deletion, conventional CD4 T cells, and Treg precursors (24). Appropriately strong or intermediate self-reactivity, together with CD28 signaling, biases cells toward Treg precursor formation (24). Consistent with this selection logic, Tregs are preferentially selected for self-recognizing specificities, resulting in a mature Treg repertoire that is largely distinct from conventional CD4 T cells (26). During this stage, Treg super-enhancers show augmented activation and several Treg signature genes begin to be expressed, while *Foxp3* and the full Treg-specific DNA hypomethylation pattern are not yet established (23). Common γ-chain cytokines, especially IL-2 acting via STAT5 (with contribution from IL-15), then drive precursor maturation to Foxp3^+^ cells (24, 27). In parallel, Treg super-enhancers become fully activated and the TSDR progressively becomes fully demethylated. Together, these changes culminate in stable nTregs, defined as CD25^hi^Foxp3^+^, which then exit the thymus with a stable Treg transcriptional and epigenetic program (22, 23) (**Figure 1**).

**Figure 1:**
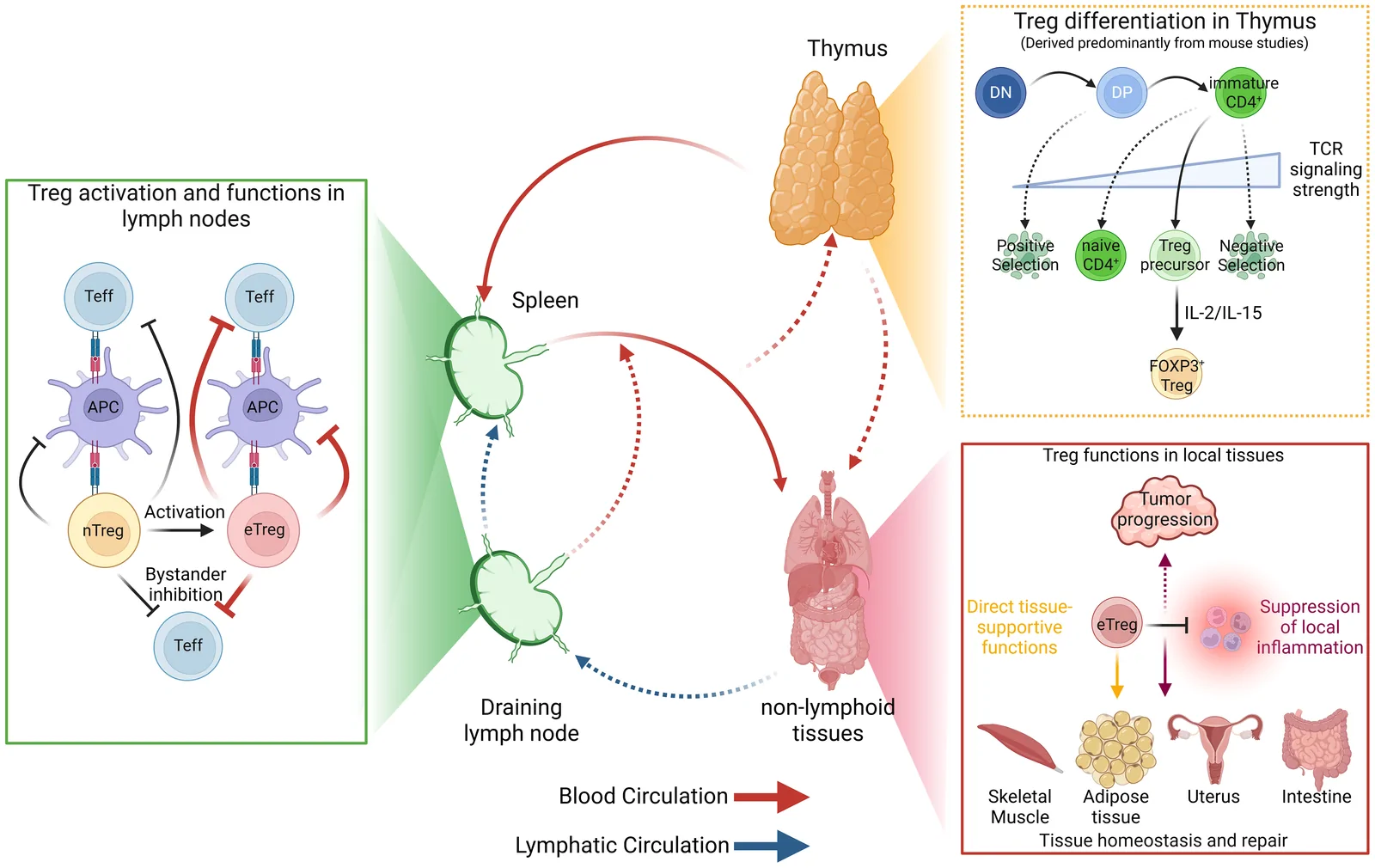
Conceptual model of human Treg development, activation, trafficking, and tissue adaptation. FOXP3^+^ Tregs develop in the thymus, enter the peripheral circulation, and recirculate through secondary lymphoid organs, where they regulate APC–Teff interactions and acquire activation-associated, tissue-homing programs. Tregs can subsequently accumulate in non-lymphoid tissues and tumors, where they adapt to local cues and mediate context-dependent immune regulation. Tissue Tregs may also contribute to tissue homeostasis and repair, whereas tumor-associated Tregs can suppress local antitumor immunity. Detailed thymic Treg developmental mechanisms and selected Treg trafficking pathways are established predominantly in mouse models and are indicated by dotted arrows. APC, antigen-presenting cell; Teff, effector T cell; nTreg, naïve regulatory T cell; eTreg, effector regulatory T cell.

### 2.2 Treg activation in secondary lymphoid organs and homing to non-lymphoid tissues

After exiting the thymus, nTregs circulate in the blood and recirculate through secondary lymphoid organs (SLOs), where they can acquire effector-associated programs following antigenic stimulation. In human peripheral blood, CD45RA^+^ nTregs are relatively enriched for the lymphoid-homing receptor CCR7, consistent with preferential SLO recirculation, although their trafficking routes and local interactions cannot be directly tracked with the resolution achievable in experimental animal models (15, 16, 28, 29). The detailed anatomical and mechanistic basis of this transition has been defined predominantly in mouse models. There, CCR7 promotes localization of naïve-like Tregs to T-cell zones and access to local IL-2 signals (30, 31). Upon recognition of self-peptide-MHC II on antigen-presenting cells (APCs), principally dendritic cells (DCs), together with costimulation and IL-2, nTregs are activated into eTregs, acquiring increased proliferative capacity and augmented suppressive machinery (22, 23) (**Figure 1**). Beyond interacting with DCs alone, high CD25 and self-reactive TCRs on Tregs facilitate their co-localization with both DCs and IL-2-secreting self-reactive Teffs, enabling direct suppression at the priming site (8, 32, 33). At the transcriptional level, this activation step is accompanied by induction of an effector-associated regulatory network (often discussed in terms of BATF/IRF4/GATA3-linked programs), which supports eTreg maturation and non-lymphoid tissue adaptation, whereas the resting identity of nTreg is reinforced by repressors of activation programs such as BACH2 and TCF1 (34). Related activated transcriptional states are observed in human peripheral-blood, tissue, and tumor Tregs, and IRF4 has been shown to support effector-Treg differentiation and suppressive function in human cancer (35). Concordantly, activation-linked effector wiring in eTregs is tightly coupled to TCR signal integration through Ca²^+^-dependent downstream programs, as shown in mice (36, 37).

Following activation, eTregs can remain within lymphoid tissues to regulate ongoing priming or migrate to non-lymphoid sites. In mice, activated eTregs exit the T cell zone and upregulate chemokine receptors and adhesion molecules associated with non-lymphoid tissue homing (38). During priming, dendritic cells can provide not only cognate antigen but also local environmental cues that imprint tissue-tropic programs on Tregs, analogous to the tissue-imprinting processes described for conventional T cells (38–40). In humans, activated Tregs with tissue-homing and tissue-residency-associated programs are observed in peripheral tissues and tumors, including populations expressing chemokine receptors and integrins associated with site-specific localization (41–43). eTregs can subsequently circulate through blood and accumulate in non-lymphoid tissues, where they acquire tissue-adapted regulatory programs and exert local immunoregulatory functions (**Figure 1**). A recent model in mice also proposes a pan-tissue migratory Treg pool with shared TCR clonality and a conserved residency program, adding complexity to how Tregs act during circulation. This pool can enter multiple (non-gut) non-lymphoid tissues at a low rate and be retained upon antigen recognition, enabling percolation across multiple tissues (44).

### 2.3 Re-entry into draining lymph nodes, secondary non-lymphoid tissues and potentially the thymus

In mouse models, as part of lymphatic trafficking, a fraction of tissue eTregs exit inflamed non-lymphoid tissues through afferent lymphatics and enter draining lymph nodes (dLNs), where they continue to suppress alloimmune responses by inhibiting the activity and accumulation of Teffs and DCs (45). After reaching dLNs, some eTregs can re-enter the blood and re-localize to distant non-lymphoid sites, demonstrated for trafficking from the large-intestine lamina propria to distal locations (46), and consistent with the pan-tissue, percolating Treg pool model discussed above (44). In addition, mouse studies have shown that peripheral antigen-experienced eTregs, but not nTregs, can sometimes recirculate to the adult thymus and suppress the development of new Treg precursors, providing a feedback layer to central tolerance (47) (**Figure 1**).

### 2.4 Expanding the functional Treg pool via Th-like specialization

Beyond trafficking programs that position Tregs at the right sites, additional mechanisms expand the functional Treg pool. eTregs can sense and integrate local metabolic and inflammatory cues to tailor suppressive activity to the tissue context. In many circumstances, these cues parallel those that drive Teff polarization. Consequently, eTregs upregulate matching transcriptional modules and chemokine receptors while maintaining suppressive identity, differentiating into specialized Th-like Tregs—populations that are also detectable in human peripheral blood (48, 49). Mechanistic studies, largely in mouse models, have defined the environmental and transcriptional cues that promote these specialized programs (8, 19). By coordinating tissue-homing receptors with context-matched regulatory programs, specialized Tregs can co-localize with relevant effector responses and provide targeted suppression. The major specialized Treg subsets are summarized below (**Figure 2**).

**Figure 2:**
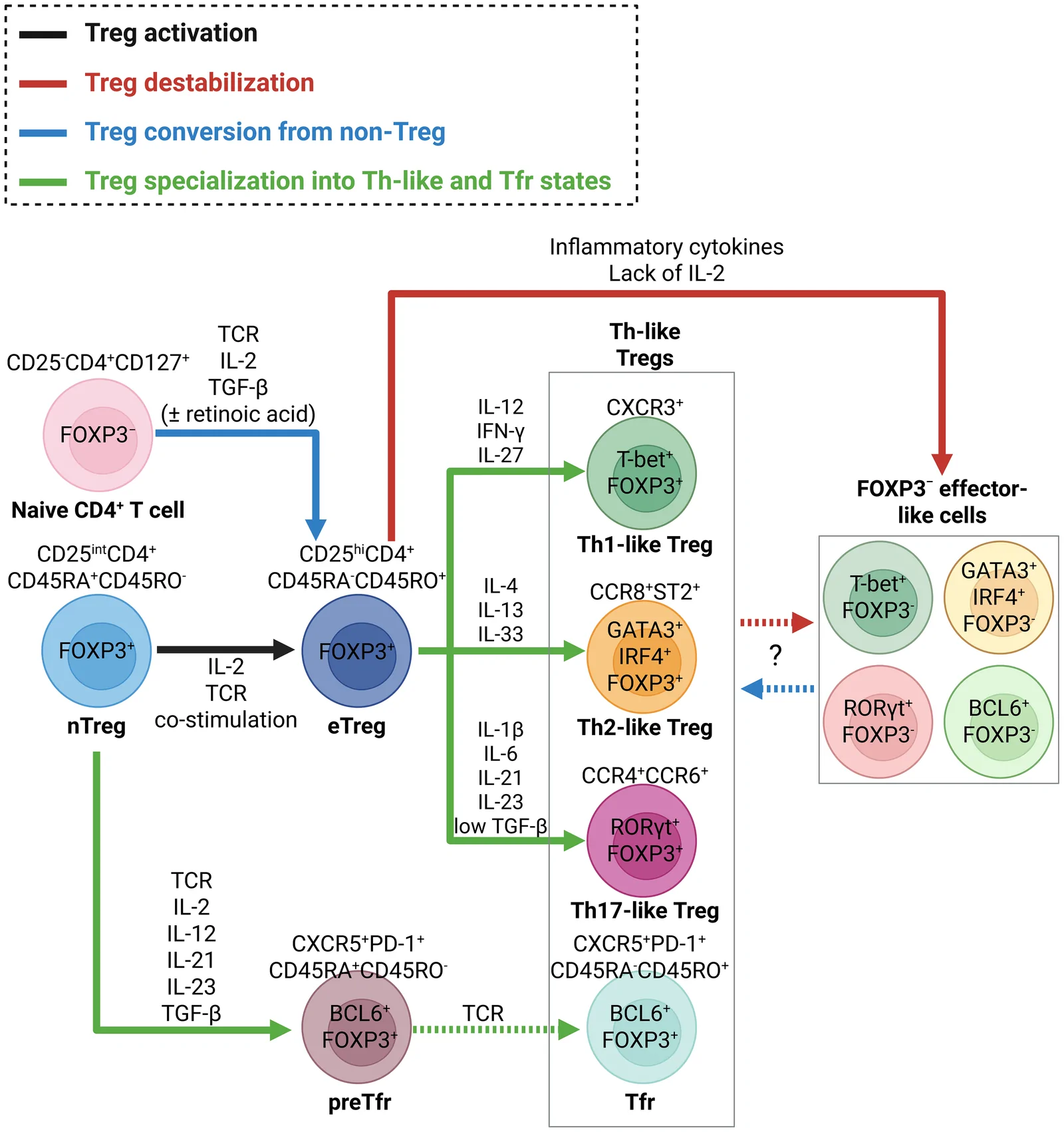
Conceptual model of the major trajectories of human Treg differentiation and plasticity. nTregs can acquire an activated eTreg state following antigen recognition, costimulation, and IL-2 signaling. FOXP3^−^ conventional CD4^+^ T cells can generate eTregs *in vivo* or *in vitro* under tolerogenic conditions, including TCR stimulation with TGF-β and IL-2, with retinoic acid providing additional support in some settings. eTregs can further specialize into Th1-like, Th2-like, Th17-like, or Tfr states that couple context-specific transcriptional programs with appropriate homing receptors; Tfr cells are typically generated during early nTreg activation. Under destabilizing conditions, including inflammatory cytokine exposure, lymphopenia, or limited IL-2 signaling, some Tregs may lose FOXP3 and acquire effector-like properties. Differentiated effector/memory conventional CD4^+^ T cells can also be reprogrammed *ex vivo* into stable, suppressive FOXP3^+^ Treg-like cells by reinforced tolerogenic signaling, reduced CD28 costimulation, and/or epigenetic modulation. Several differentiation cues shown are principally defined in mice; although corresponding human populations and phenotypic programs have been identified, their developmental relationships remain incompletely resolved. Dotted arrows indicate proposed or incompletely established transitions in humans. nTreg, naïve regulatory T cell; eTreg, effector regulatory T cell; Tfr, T follicular regulatory cell.

#### 2.4.1 Th1-like (T-bet^+^, CXCR3^+^)

Th1-like Tregs express T-bet and CXCR3, enabling their localization to CXCL9-, CXCL10-, and CXCL11-rich sites of type 1 inflammation and facilitating regulation of Th1 and CD8^+^ T-cell responses. In mice, T-bet expression in Tregs is induced during type 1 inflammation and is required for optimal control of Th1-associated immune responses (50, 51). IL-12, alone or together with IFN-γ and IL-27, can promote this program in experimental systems (52). In humans, T-bet^+^CXCR3^+^FOXP3^+^ Tregs have been detected in peripheral blood and in autoimmune disease, and human Tregs can acquire Th1-associated features under inflammatory conditions (48, 53, 54). Functionally, Th1-like Tregs help restrain type-1 inflammation in infection (51, 55), type 1 diabetes (56), and tumors (55, 57, 58). However, under certain conditions human FOXP3^+^ Th1-like Tregs may produce IFN-γ and show reduced suppressive activity, as reported in multiple sclerosis and type 1 diabetes (53, 54). Such findings indicate context-dependent functional impairment rather than establishing that Th1-like specialization intrinsically represents lineage destabilization.

#### 2.4.2 Th17-like (RORγt^+^, CCR6^+^)

The differentiation cues for Tregs and Th17 cells substantially overlap, with both requiring TGF-β, creating a mechanistic basis for interconversion (59). Consistent with this, FOXP3^+^ cells bearing Th17 features—active STAT3, RORγt and CCR6 (often CCR4), and sometimes low-level IL-17—are observed in mice and humans (48, 49, 60–62). In mouse models, FOXP3^+^RORγt^+^ Th17-like Treg cells are enriched at mucosal barriers, especially the intestine, where microbial, infectious, and inflammatory signals shape their development. These cells mainly modulate Th17 responses (63–65), while in some contexts also modulate Th1 and Th2 responses (64, 66, 67). Beyond mucosal barriers, a recent mouse study showed that uterine Treg specialization can also include RORγt^+^ Tregs that restrain local Th17 programs in that niche, highlighting that Th17-like regulatory adaptation extends to reproductive immune environments (68). In *in vitro* experimental systems, Th17-like Tregs can be induced by different combinations of IL-1β, -6, -21, and -23, with autocrine low-level TGF-β being indispensable, whereas high exogenous TGF-β prevents this program (61, 62, 69), supporting a TGF-β signal-strength model for the Treg–Th17 axis (59, 61). Although RORγt^+^FOXP3^+^ Tregs can retain suppressive function in homeostatic settings, dysregulated Th17-associated programs may be accompanied by inflammatory cytokine production and impaired regulatory activity. Such phenomena have been demonstrated in mouse models of autoimmune arthritis (70), while IL-17-producing or RORγt^+^ FOXP3^+^ populations have been described in human inflammatory bowel disease and colon cancer (71, 72).

#### 2.4.3 Th2-like (GATA3^+^, IRF4^+^, CCR8^+^, ST2^+^)

Th2-like Tregs are less well defined than Th1- and Th17-like counterparts, but are commonly characterized by GATA3 and IRF4 expression and are implicated in selective control of type 2 immunity. In mouse models, IRF4 and GATA3 contribute to Treg-mediated control of Th2 responses and maintenance of Treg function during inflammation (73, 74). These GATA3- and IRF4-expressing Th2-like Tregs are enriched for CCR8 and ST2 (IL33R), surface markers for Th2 cells (75, 76). In human peripheral blood and tumors, Th2-like Tregs with GATA3-, IRF4-, and CCR8-associated features have been identified, and IRF4 has been shown to promote effector-Treg differentiation and suppressive function in human cancer (35, 49). In mice, *GATA3* can be directly induced by TCR and IL-2 signaling in Treg, which can be dampened by IL-12, IL-27, and IL-6, and can be enhanced by IL-4 (74). Mechanistically, GATA3 can directly influence *FOXP3* transcription and cooperate with FOXP3 to support type 2 regulatory programs, helping prevent destabilization toward pathogenic Th17 states (77, 78). Th2-like Tregs can be polarized *in vitro* from established Tregs by IL-4 + IL-13 (79) or IL-33 (76) (**Figure 2**). Notably, although IL-4 can reinforce Th2-like features in established Tregs, it can inhibit iTreg induction from naïve CD4^+^ T cells, potentially via HDAC9-dependent mechanisms (80, 81). While Th2-like Tregs contribute to protection in type-2 contexts, they may be comparatively prone to functional erosion or pathogenic roles. Th2-like Tregs appear relatively viable and enriched in human melanoma and colorectal tumors compared with other Th-like subsets (49). In mouse models, IL-4-producing FOXP3^+^ Tregs have been linked to impaired oral tolerance and food allergy, whereas Tregs can acquire Th2-like effector features with reduced suppressive function during RSV-induced lung inflammation (79, 82).

#### 2.4.4 Tfr (CXCR5^+^)

T-follicular regulatory cells (Tfrs) are a specialized FOXP3^+^ Treg subset characterized by CXCR5 expression and variable expression of PD-1 and BCL6. Tfrs acquire a follicular homing program to enter B-cell areas and modulate T follicular helper (Tfh)-dependent germinal center (GC) responses (83). In mouse models, Tfrs are generated predominantly during the initial activation and priming of naïve-like Tregs, rather than arising exclusively from established eTregs (83, 84). They are characterized by downregulation of CD25 during maturation in the GC (85). This may complicate discrimination from Tfh-like cells that transiently express FOXP3 in mouse GCs (86, 87). In addition to GC-resident Tfrs, circulating Tfrs (cTfrs) have been identified in humans and typically express higher CD25 and lower PD-1 than GC-resident Tfrs, reflecting their intermediate activation stage (83, 88). It was recently found that 30 to 50% of human cTfrs are at a naïve CD45RA^+^CD45RO^−^ stage (preTfr). These cells are transcriptionally closest to nTregs, are expandable *in vitro*, and upon stimulation acquire Tfr-associated suppressive features (88). In the same study, “Tfr-promoting conditions” (TPC), consisting of IL-2, IL-12, IL-21, IL-23, and TGF-β, promoted differentiation of human nTregs toward a Tfr-like state *in vitro* (88). In humans, an altered Tfh/Tfr ratio has been associated with autoimmune dysregulation in rheumatoid arthritis, systemic lupus erythematosus (SLE), Sjögren’s syndrome, multiple sclerosis, and type 1 diabetes (89).

### 2.5 Expanding the Treg avidity and TCR repertoire pool through conversion from non-Tregs

In addition to thymic origin (tTreg), the avidity and TCR repertoire of Tregs can be continually supplemented in the periphery through the differentiation of pTregs from FOXP3^−^ conventional CD4^+^ T cells (Tconvs; **Figure 2**). In mouse models, pTreg generation is most prominently associated with mucosal tissues, particularly the gut, where naïve (and more rarely antigen-experienced) CD4^+^ T cells encounter self and non-self-antigens, including diet- and microbiota-derived antigens, in a context shaped by specialized antigen-presenting cells (APCs) and local metabolites (34, 90–93).

Mechanistically, foundational *in vitro* work in mice established that TCR stimulation plus TGF-β and IL-2 can induce *Foxp3* expression in naïve CD4^+^ T cells, generating *in vitro*–induced iTregs (94). A corresponding approach has also been demonstrated in human naïve CD4^+^ T cells, in which TCR stimulation with TGF-β and IL-2, often supplemented with agents such as all-trans retinoic acid, generates CD25^+^FOXP3^+^ iTreg-like cells with regulatory activity (95–97). Notably, because TGF-β can also participate in Th17 differentiation, the surrounding cytokine environment, particularly IL-2 (acting via STAT5) versus IL-6/IL-21 (acting via STAT3), strongly influences whether TGF-β drives a FOXP3- or RORγt-dominated program (59). In addition to the key cytokines, multiple metabolites can further bias this axis, including aryl hydrocarbon receptor (AHR) ligands, selected bile acid metabolites, and sphinganine (98–100). In mouse models, subsequent studies implicated additional permissive inputs for pTreg differentiation, including retinoic acid, PD-L1/PD-1 signaling, and reduced CD28 costimulation (101–104). This conversion can also be supported by other cell types, such as mesenchymal stem cells (MSCs) (105) and CD103^+^ gut-associated dendritic cells (101). More recently, several breakthroughs in mouse models have identified candidate APCs specialized for gut pTreg induction that express RORγt, including MHCII^+^ group 3 innate lymphoid cells (ILC3) (106), Janus cells (JC) (107), integrin αvβ8^+^ type IV Thetis cells (TC IV) (107, 108), and PRDM16^+^RORγt^+^ tolerizing dendritic cells (93).

Conversion of effector/memory Tconvs into FOXP3^+^ Tregs appears less common than naïve-to-pTreg induction, consistent with the idea that differentiated effector programs are stabilized and more resistant to lineage reprogramming (109). Nonetheless, such transitions have been documented under reinforced tolerogenic conditions. In mice, examples include Th2-to-Treg redifferentiation driven by TGF-β together with all-trans retinoic acid and rapamycin (110), continuous oral antigen exposure promoting antigen-specific, tissue-adapted pTregs (111), and an IFN-γ fate-mapping approach in tumors showing that Th1 cells can acquire FOXP3 and differentiate into Th1-like pTregs in the tumor microenvironment in a TGF-β-dependent manner (112). Extending TGF-β-based strategies, co-delivery of tolerogenic cytokine signals can induce stable pTregs *in vivo* even in inflammatory settings: an IL-2–TGF-β surrogate co-agonist that couples IL-2/STAT5 and TGF-β/SMAD2/3 signaling selectively in IL-2R–expressing T cells induced antigen-specific, functional pTregs and promoted immune tolerance across models including intestinal inflammation and autoimmune neuroinflammation (113). Beyond cytokine pairing, pharmacologic rewiring can also promote *de novo* FOXP3 acquisition with improved stability: CDK8/19 inhibition can drive antigen-specific FOXP3^+^ Tregs via STAT5-biased signaling independently of exogenous TGF-β, and co-administration with antigen immunization generated suppressive FOXP3^+^ Tregs that controlled inflammation *in vivo* (114). Notably, deprivation of CD28 costimulation during TGF-β/IL-2-driven iTreg induction enables even effector/memory Tconvs to acquire epigenetically stabilized FOXP3 expression (104).

In humans, early evidence for direct effector-to-FOXP3^+^ conversion included PD-1-associated conversion of Th1 cells toward a regulatory phenotype (115). More recently, three studies have shown that human effector/memory CD4^+^ Tconvs can be converted *ex vivo* into functionally stabilized FOXP3^+^ Tregs by jointly enforcing *FOXP3* transcription and installing a Treg-type epigenome (116–118). Two studies established “stable/functional iTreg” protocols that combine CDK8/19 inhibition (e.g., senexin A or AS2863619) with IL-2 (± TGF-β) while withholding CD28 costimulation (e.g., omitting anti-CD28 or using CTLA4-Ig), using iterative stimulation followed by IL-2-only resting and expansion cycles. These protocols converted mouse and human effector/memory CD4^+^ Tconvs, including Th1/Th2/Th17-polarized cells, into FOXP3^hi^, TSDR-demethylated, CTLA-4^hi^ Tregs with enhanced stability and suppressive function *in vivo*, including disease-/antigen-specific suppression in pemphigus vulgaris and in models of colitis and GvHD (116, 117). A third mouse study showed that epigenetic reprogramming of autoreactive Teff using a TGF-β/retinoic acid/vitamin C program with sustained restimulation can drive demethylation of core Treg identity loci (including *Foxp3* TSDR) to generate lineage-stable effector-reprogrammed Tregs (ER-Tregs) that retain autoantigen specificity and can arrest established experimental autoimmune encephalomyelitis (EAE) (118). Conceptually, these approaches are attractive because they could repurpose pathogenic or tissue-/tumor-reactive effector repertoires into regulatory repertoires. In addition to FOXP3^+^ cells, in many tolerance settings the dominant outcomes of conversion from effector Tconvs instead map to FOXP3^−^ regulatory lineages, for example Tr1 cells (119, 120), further expanding the available suppressive capacity.

### 2.6 Treg destabilization and exTreg formation

The plasticity of Tregs enables functional specialization but can also, in some settings, be associated with destabilization into pathogenic effector-like cells, termed exTregs (**Figure 2**). This phenotype is best characterized in mouse Treg fate-tracking models, for example *Foxp3^EGFP-CRE^* x *Rosa26^YFP^*, where exTregs are detected as YFP^+^GFP^-^ (70, 121). Under inflammation or lymphopenia, a minority of Tregs lose FOXP3, acquire effector programs, produce pro-inflammatory cytokines, and exacerbate disease (70, 121). IL-2 signaling safeguards lineage stability, acting at the *Foxp3* TSDR locus (122, 123). Because Tregs are enriched for self-reactive TCR specificities, exTregs arising from committed Tregs may retain autoreactive potential and contribute to autoimmune pathology. Human exTregs are difficult to track prospectively, although a recent study suggested CD3^+^CD4^+^CD16^+^CD56^+^ cytotoxic cells as a putative human exTreg population (124). In patients with IPEX (immune dysregulation, polyendocrinopathy, enteropathy, X-linked) syndrome caused by FOXP3 deficiency, FOXP3^−^ effector T cells with a demethylated TSDR have been interpreted as exTreg-like cells with pathogenic activity (125, 126).

Different models propose distinct origins for exTregs. One model posits that most apparent exTregs arise from transiently activated Tconv cells that briefly express *FOXP3*, or from cells skewed under extreme cell stress such as lymphopenia (127, 128). It also highlights a pre-existing minor population (often Helios^−^ with incomplete Treg-specific DNA demethylation) that is more prone to apparent conversion, whereas epigenetically committed Tregs with maintained TSDR demethylation are largely stable and, even if they transiently downregulate *FOXP3*, can reacquire *FOXP3* expression and suppressive function when destabilizing cues abate (7, 129). In contrast, another model suggests that, during inflammatory responses, a substantial fraction of established antigen-specific Tregs with stable features (Foxp3^hi^, CD25^hi^, demethylated TSDR) can still downregulate FOXP3 and acquire effector characteristics and pathogenic potential (123). Supporting this, prolonged exposure to inflammatory cytokines can drive mouse and human Tregs, including nTregs, to lose FOXP3 and suppressive function while producing inflammatory cytokines (39, 54, 130). Although mechanisms of Treg instability remain incompletely defined, it is broadly accepted that *in vitro*–induced iTregs and peripherally induced pTregs often lack full Treg-specific epigenetic features and are more prone to instability than tTregs and nTregs (18, 34, 131). Importantly, clinical studies of adoptive transfer of highly purified CD25^+^CD127^low^ Tregs have not shown pathogenic conversion, supporting the use of unedited Tregs for therapy when products meet stability criteria (132, 133).

Two open questions remain to better clarify the exTreg origin. First, because DNA methylation patterns are propagated during replication by the DNMT1/UHRF1 machinery and reversed via Ten-eleven translocation (TET) dioxygenases (134, 135), it is not surprising that DNMT1/UHRF1 and TET proteins have been implicated in Treg lineage maintenance (136–138). It is important to determine whether Treg-specific demethylation can be perturbed, thereby destabilizing even “stable” Tregs. Second, although Th-like Tregs are FOXP3^+^ and remain immunosuppressive in homeostasis, with only limited evidence of reduced suppressive function in certain contexts (53), it remains unclear whether Th-like Tregs are an intermediate stage before exTreg formation.

## 3 Who they are: delineating naïve and effector Treg

As discussed above, Tregs traverse multiple activation and tissue-adaptation states with distinct functions, stability constraints, and trafficking behaviors. Therefore, accurately distinguishing these states is essential for interpreting human datasets and designing interventions. This section focuses on delineating naïve (nTreg) and effector (eTreg) populations, as these two major categories are relatively tractable to distinguish, widely used across preclinical and clinical studies, and differ substantially in phenotype, function, and therapeutic utility.

### 3.1 Surface markers to distinguish nTreg and eTreg

#### 3.1.1 Classic markers

Treg origin (tTreg vs. pTreg) can be clearly defined in mice using the transcription factor Helios (139) and a surface protein neuropilin 1 (Nrp1) (140), but these markers do not reliably distinguish tTregs from pTregs in humans (141). CD31 (PECAM-1) is a recent thymic emigrant (RTE) marker that is highly enriched in human nTreg relative to eTreg. Although the overall number of CD45RA^+^CD31^+^ Tregs declines markedly with age, the proportion of CD31^+^ cells within the nTreg population remains relatively stable (15, 29). A recent study identified GPA33, a transmembrane receptor of the Ig-domain family, as a surface marker for nTregs, and suggested that it may mark Tregs of thymic origin (142).

Treg activation stage (nTreg versus eTreg) is commonly delineated in mice by CD44/CD62L (L-selectin) (143) and in humans by CD45RA/CD45RO (15, 16). Consistent with this biology, *ex vivo* expansion of human naïve CD45RA^+^ Tregs with CD3 activation converts them to activated CD45RO^+^ Tregs while maintaining lineage stability and robust suppressive function (29, 144). In human blood, the CD45RA^+^CD45RO^-^ CD4^+^CD25^+^ Treg population is relatively pure, whereas the CD45RA^-^CD45RO^+^ CD4^+^CD25^+^ population appears to be a mix of stable eTreg and unstable pro-inflammatory cells (17). This is partially because when human conventional T cells are activated, they can temporarily upregulate CD25, FOXP3 (145), and CTLA4 (146), creating difficulties in distinguishing eTregs from activated conventional cells. At the same time, eTregs tend to upregulate CD25 and FOXP3 during activation (17, 29). Consistent with this, further work segregated CD4^+^CD25^+^ cells into three fractions: Fraction I (Fr. I) CD45RA^+^FOXP3^int^/CD25^int^, a relatively pure nTreg population, Fr. II CD45RA^−^FOXP3^high^/CD25^high^, a relatively pure eTreg population, and Fr.III CD45RA^-^FOXP3^lo^/CD25^lo^, a mixed population that includes non-Tregs and a minority of Tregs (17, 18). In these definitions FOXP3 and CD25 levels run in parallel, with CD25 serving as the surface stand-in for FOXP3. The distinction matters in practice: FOXP3 staining requires fixation and permeabilization, so FOXP3-based gates can only be used for analysis, whereas CD45RA/CD25 combinations can be applied to viable cells and are therefore used to isolate Tregs for functional assays, epigenetic analysis and cell manufacturing. The substitution is approximate, because CD25 level tracks FOXP3 level rather than reporting it directly. FOXP3 combined with HELIOS can identify stable Treg populations, but both markers require intracellular staining (147). As a practical surface marker, CD127 (IL-7Rα) level inversely correlates with FOXP3 (148), though it can be transiently modulated in Tconv (temporary downregulation) (145) and Tregs (temporary upregulation) (149) upon activation. Incorporating CD127 identifies a CD127^−^ eTreg subpopulation within Fr.III, which is almost proteomically indistinguishable from Fr.II eTreg but less stable than Fr.II eTreg (higher TSDR methylation and higher levels of inflammatory cytokine production) (150) (**Table 2**).

**Table 2:** Practical surface markers combinations for isolating Treg components from human blood.

Starting population	Ref	Sub-Fraction		
CD4^+^ T cells (peripheral blood)	(17)	**Fraction I (nTreg)**	**Fraction II (eTreg)**	**Fraction III (a mix of eTreg and non-Treg)**
		CD45RA^+^CD25^int^	CD45RA^−^CD25^hi^	CD45RA^-^CD25^lo^
Fraction III (CD45RA^−^CD25^lo^)	(150)	**eTreg**	**non-Treg**	
		CD127^-^ or CD127^+^CD49d^−^CCR4^+^	CD127^+^CD49d^+^CCR4^-^ or CD127^+^CD49d^−^CCR4^-^	
	(83, 151)	**eTreg**	**non-Treg**	**cTfr**
		CD49d^−^CCR4^+^CXCR5^−^	CD49d^+^CCR4^−^CXCR5^−^	CD49d^−^CCR4^−^CXCR5^+^
CD25^+^CD4^+^ CD45RA^−^CD127^lo^	(152)	**eTreg**	**nonsuppressive FOXP3^+^ T cells**	
		CD15s^+^	CD15s^-^	

nTreg, naïve regulatory T cell; eTreg, effector regulatory T cell; non-Treg, non-regulatory T cell; cTfr, circulating T follicular regulatory cell.

#### 3.1.2 Costimulatory and coinhibitory molecules

Costimulatory and coinhibitory molecules are commonly associated with activated and effector T-cell states (153). These molecules are frequently upregulated in eTregs, particularly in highly activated tissue-resident and tumor-infiltrating eTregs. Examples include the coinhibitory molecules CTLA4, PD1 (15, 17, 154), TIGIT (155), LAG3 (156), TIM3 (157), and CD39 (ENTPD1) (158), as well as the costimulatory molecules ICOS (17), CD27 (TNFRSF7) (159, 160), 4-1BB (TNFRSF9) (161), and GITR (TNFRSF18) (163, 164) (**Table 3**).

**Table 3:** Summary of potential surface markers for human naïve and effector Tregs.

Category	Marker/axis	nTreg	eTreg	Functions/notes	References
tTreg markers	CD31 (PECAM-1)	Enriched	Reduced	Marker of recent thymic emigrants (RTE)	(15, 29)
	GPA33	Enriched	Reduced	Potentially defines human tTreg	(142)
Classic Treg subtyping markers	CD45RA/CD45RO	CD45RA^+^ CD45RO^−^	CD45RA^−^ CD45RO^+^	CD45RA^−^ CD45RO^+^ gate of eTreg can be contaminated by activated Tconv unless combined with FOXP3/CD25/CD127	(15, 16, 17, 29, 145)
	CD25 (IL-2RA)	Intermediate	High	Useful for Treg enrichment, but CD25 alone does not define Treg or eTreg	(1, 17, 145)
	CD127 (IL-7Rα)	Low/-	Low/-	Practical surface complement to CD25/FOXP3; Can transiently shift in both Tregs and Tconv with activation	(148, 145, 149)
Coinhibitory molecules	CTLA-4	Intermediate	High	Intracellular staining is more sensitive	(17, 8, 146)
	PD-1	Low to intermediate	High	Activation-dependent; marks cell state rather than lineage	(15, 17, 154)
	TIGIT	Low	High	Activation-dependent; marks cell state rather than lineage	(155)
	LAG-3	Low	High	Activation-dependent; surface level underestimates expression because LAG3 is rapidly internalised and shed	(156)
	TIM-3 (HAVCR2)	Low	High	Activation-dependent; marks cell state rather than lineage	(157)
	CD39 (ENTPD1)	Low	High	Often paired with CD73 in mice	(158, 8)
Costimulatory molecules	ICOS	Low	High	Activation-dependent; marks cell state rather than lineage	(17)
	CD27 (TNFRSF7)	Low	High	Activation-dependent; marks cell state rather than lineage	(160, 159)
	4-1BB (TNFRSF9)	Low	High	Activation-dependent; marks cell state rather than lineage	(161)
	GITR (TNFRSF18)	Low	High	Activation-dependent; marks cell state rather than lineage	(163, 164)
Homing molecules	CCR7	High	Low/variable	Tracks SLO recirculation; Some eTreg subsets remain CCR7^+^ (sometimes called “central memory-like” eTreg).	(28, 31, 165, 166)
	CXCR3	Low	Subset-enriched	Higher in Th1-like eTreg	(50, 48, 51)
	CCR8	Low	Subset-enriched	Higher in Th2-like eTreg and some tumor-enriched -eTreg	(49, 167, 168, 169)
	ST2 (IL1RL1; IL-33R)	Low	Subset-enriched	Higher in Th2-like eTreg	(76, 35, 75)
	CCR4	Low	Subset-enriched	Higher in Th17-like eTreg and some tumor-enriched -eTreg	(170, 48, 150, 41)
	CCR6	Low	Subset-enriched	Higher in Th17-like eTreg	(170, 48, 171)
	CXCR5 (Tfr/cTfr)	Low	Subset-enriched	Higher in Tfr; Circulating cTfr can include CD45RA^+^ “pre-Tfr” states	(83, 88, 151)
	CCR5	Low	Subset-enriched	Higher in some tumor-enriched -eTreg	(172, 173)
	CD49d (integrin α4)	Low	Subset-enriched	In blood, helps dissect Fr. III heterogeneity with CCR4/CXCR5 (non-Treg vs Treg-like fractions)	(150, 151, 83)
	integrin β7	Low	Subset-enriched	Higher in gut-homing eTreg	(42, 174)
	CD103 (integrin αE)	Low	Subset-enriched	Higher in gut-homing eTreg and some tumor enriched eTreg	(28, 175, 43)
Other molecules	HLA-DR (human)	Low/-	High	Classic human activated T cell marker; Correlates with increased suppressive capacity	(15, 176)
	CD38 (human)	Low	High	Can be used with HLA-DR to stratify -eTreg- states	(177, 178, 179)
	LAP	Low	High	LAP is part of the precursor protein of TGF-β	(15)
	GARP (LRRC32)	Low	High	Functions in docking and activating latent TGF-β	(180, 15)

#### 3.1.3 Homing molecules in tissue-resident or tumor-infiltrating Tregs

Many chemokine receptors and adhesion molecules are specifically expressed by distinct Treg subsets, directing their localization and function. A detailed dissection of Fr. III suggests that CD127^−^CD25^int^CD45RA^−^ cells can be further divided into three groups based on CD49d (integrin α4) and CCR4 expression. CD49d^−^CCR4^+^ cells produce IL-2, but not other inflammatory cytokines, and are most similar to Fr. II eTregs, whereas the CD49d^+^ fraction expresses inflammatory cytokines (150) (**Table 2**). The incorporation of the Tfr marker CXCR5 with CD49d and CCR4 can stratify Fr. III into CD49d^+^CXCR5^−^CCR4^−^ non-Tregs, CD49d^−^CXCR5^+^CCR4^−^ circulating T follicular regulatory (cTfr) cells, and CD49d^−^CXCR5^−^CCR4^+^ Tregs (83, 151) (**Table 2**). Additionally, within the CD45RA^−^CD127^low^CD25^+^CD4^+^ eTreg population, CD15s (sialyl Lewis x) was shown to define a CD15s^+^ suppressive population with high FOXP3 expression and a CD15s^-^ non-suppressive population with low FOXP3 expression, which may help exclude non-Treg cells (152) (**Table 2**). In mice, CCR7 promotes nTreg cell recirculation through secondary lymphoid organs (28, 31). In humans, CCR7 is enriched in CD45RA^+^ nTregs relative to CD45RA^−^ eTregs (15, 16). Following canonical T cell nomenclature (153), recent single-cell studies sometimes subdivide eTregs (CD25^hi^CD127^-^CD45RA^−^) into central memory (CD45RA^−^CCR7^+^) and effector memory (CD45RA^−^CCR7^−^) populations (165, 166) (**Table 3**).

As discussed above, chemokine receptors including CXCR3, CCR4, CCR8, CCR6, and CXCR5 have also been used to characterize Th-like Treg subtypes in human peripheral blood. In mice, CCR2, CCR4, CCR5, CCR6, and CCR8 are generally enriched in eTreg (CD62L^−^FoxP3^+^) (38). In humans, CCR4, CCR6, and CCR10 are enriched in CD45RO^+^ eTregs (48, 170). Among these, CCR2, CCR4, and CCR5 promote the homing of activated Tregs to inflamed sites in mice (45); CCR2 promotes eTreg accumulation within inflamed visceral adipose tissue, especially in female mice (181); and CCR2 expression on Tregs has been implicated in tumor infiltration in mouse and human tumors, especially in tumors with *MLL3* mutations (162, 182). CCR4 is important for Treg localization to skin and lung in mice (183) and is predominantly expressed by highly suppressive human eTregs in malignant melanoma (15, 41). CCR5 facilitates Treg recruitment into the tumor microenvironment, and disruption of CCR5-dependent homing impairs intratumoral Treg accumulation and inhibits tumor growth in murine pancreatic cancer and skin squamous cell carcinoma models (172, 173). In humans, more than 90% of gastric mucosal Tregs express CCR6 and further accumulate during *Helicobacter pylori* infection (171). In mice, CCR6 is required for Treg recruitment to inflamed tissues in glomerulonephritis and EAE (184, 185). CCR8^+^ Tregs constitute 30–80% of human tumor Tregs in various cancers and fewer than 10% of Tregs in other tissues, and define a highly activated Treg population in mice and humans (167, 168) (**Table 3**).

In addition to CD49d (integrin α4), other integrins are enriched in eTregs and contribute to Treg migration. CD49b (integrin α2) marks a mature eTreg population that continuously surveys the skin, vasculature, and lymphoid tissues in mice and exhibits heightened suppressive capacity; however, CD49b is present in only a small fraction of, or is undetectable on, human Tregs (186). In mice, CD103 (integrin αE) expression positively correlates with Treg activation and suppressive potency (28, 187) and has been implicated in Treg retention within gut mucosal tissue (175). Recent work identified a CD103^+^ Treg population associated with resistance to radioimmunotherapy in human glioblastoma (43). Integrins α4 and β7 are also important for Treg homing to the gut and are increased on Tregs from patients with ulcerative colitis (42). In mice, integrin β7 deficiency or blockade reduces Treg accumulation in the gut and exacerbates intestinal inflammation, without directly impairing Treg suppressive function (174) (**Table 3**).

#### 3.1.4 Other molecules

HLA-DR, a T cell activation marker, is well-established to be enriched in eTregs (15, 176). More recently, CD38, another T cell activation marker, has also been found to be enriched in immunosuppressive eTregs in cancers and inflammatory settings (177, 188–191). A recent study further subdivided human blood CD45RA^−^ eTregs into four subsets based on CD38 and HLA-DR expression: CD38^+^ HLA-DR^+^ double-positive (DP) cells were the most activated and proliferative; HLA-DR^+^ single-positive (SP) cells were skin-homing; CD38^+^ SP cells were gut-homing; whereas double-negative (DN) Tregs showed limited proliferation, similar to CD45RA^+^ nTregs (178). Among these, CD38^+^HLA-DR^lo^ cells were enriched in patients with critical COVID-19 and were suggested to represent recently activated eTregs (179) (**Table 3**).

Surface components of the TGF-β signaling pathway, including latency-associated peptide (LAP), which forms a latent complex with TGF-β, and glycoprotein A repetitions predominant (GARP), which facilitates TGF-β activation, are highly expressed on activated Tregs and contribute to suppressive function (15, 180) (**Table 3**).

### 3.2 Different Treg features in the naïve and effector subsets

#### 3.2.1 TCR repertoire diversity

nTregs generally maintain broader TCR repertoire diversity than eTregs, consistent with nTregs serving as a renewable, polyclonal reservoir and eTregs being enriched for selected clones in response to antigenic challenges. In line with this, a recent analysis of human Treg repertoires showed that CD45RA^+^ nTregs exhibit high diversity, whereas CD45RA^−^ eTregs display comparatively greater clonal expansion (166). This clonal skewing becomes more pronounced as eTregs adopt tissue-resident programs, where local antigen and inflammatory cues promote preferential accumulation of specific regulatory clones. For example, in mice, Tregs in visceral adipose tissue (VAT) (192, 193), skeletal muscle (9), and the colonic lamina propria (90, 194) show tissue-adapted transcriptional specialization together with biased TCR repertoires. In human breast cancer, tumor-infiltrating Tregs likewise display a distinct TCR repertoire and tissue-adapted phenotype (167). Similarly, specialization into Th-like Treg states may further stratify TCR repertoires within the broader eTreg pool. In a mouse model of type 1 inflammation, T-bet^+^ Th1-like Tregs possessed a distinct TCR repertoire compared with other Treg states, consistent with selective recruitment or expansion of antigen-specific clones into the Th1-like regulatory niche (51).

#### 3.2.2 Proliferative ability

In steady state, nTregs undergo measurable homeostatic proliferation driven by continual exposure to self-antigens, consistent with a constitutively poised suppressive program (15, 143). However, their turnover rate remains relatively restrained, potentially due to low-intensity tonic TCR signaling (signal 1), comparatively restricted costimulation (signal 2), and IL-2 scarcity (signal 3) in non-inflamed settings; this restrained activation state is further supported by high expression of *BACH2*, a key transcriptional repressor of Treg activation programs (195–197). In contrast, most eTregs are typically highly proliferative, often Ki-67^+^, and can show higher proliferation rates than Tconvs in *in vitro* settings (17, 29). However, this is not uniform, as a Ki-67^−^ fraction is usually detectable within eTregs (31), consistent with multiple activation states within the eTreg pool. Despite this heightened cycling, eTregs also exhibit increased susceptibility to apoptosis, which has been linked to reduced expression of pro-survival factors, including Bcl-2 and Mcl-1 (17, 31).

Functional proliferative capacity also differs depending on the stimulus context. Upon strong *in vitro* TCR stimulation, nTregs can proliferate more vigorously than pre-existing eTregs, consistent with nTregs acting as a “renewable” reservoir that can rapidly expand and differentiate when provided with adequate activation and cytokine support (17). In parallel, mechanistic work in mice suggests that, once the eTreg program is established, maintenance can become less dependent on IL-2 signaling than that of nTregs and instead relies more heavily on continuous TCR signals and ICOS-dependent costimulation (31, 198). Notably, this TCR dependence may not be uniform across all tissue-adapted endpoints: recent mouse work in the colon identified a terminally differentiated IL-10-stable eTreg population whose persistence and regulatory activity are associated with attenuated TCR signaling and partial acquisition of TCR-independent functionality, highlighting that TCR requirements can shift again at late stages of eTreg differentiation (199).

#### 3.2.3 Stability and inhibitory potency

Stability and suppressive potency are tightly linked because both require durable maintenance of a FOXP3-driven, epigenetically reinforced lineage program. Across developmental routes, naturally occurring Tregs are more committed than *in vitro*–induced Tregs, which can express high levels of FOXP3 protein yet fail to fully install the TSDR-demethylation pattern (7, 200). Notably, Tregs induced *in vivo*, in a setting resembling pTreg differentiation, progressively acquire Treg-specific DNA demethylation over time, consistent with the installation of this epigenetic program during pTreg differentiation (7). Within naturally occurring Treg pools, tTregs are often considered more stable than pTregs, with higher TSDR-demethylation levels, potentially reflecting their distinct developmental wiring. For example, tTreg development involves Satb1-mediated super-enhancer priming, whereas pTreg formation is relatively Satb1-independent and can even be facilitated by Satb1 deficiency (23, 25). For naïve versus effector human Tregs, a common view is that Fr. I nTregs and Fr. II eTregs in human blood are both stable populations, with similar TSDR-demethylation levels, which may be slightly higher in Fr. II eTregs and could reflect an increased requirement in activated eTregs to sustain high FOXP3 expression. In contrast, Fr. III shows lower overall TSDR demethylation, consistent with its mixed composition (17, 122). At the same time, CD45RA^+^ Tregs are generally considered more stable than CD45RA^−^ Tregs because they are enriched for thymus-derived Tregs and because the CD45RA^+^ gate in practice yields a higher-purity Treg population than CD45RA^−^ effector gates (17, 133).

Regarding inhibitory potency, Th-like Tregs can exhibit enhanced context-matched suppression that mirrors effector Th programs (48). In contrast, in standard *in vitro* non-specific suppression assays, nTregs and eTregs show similar suppressive capacity (29), and recently developed single-cell functional profiling further supports substantial heterogeneity and overlap in per-cell suppressive capacity across these two human Treg states (179). This similarity is expected because these assays are likely governed by pan-inhibitory mechanisms, such as IL-2 deprivation and inhibitory cytokines production (8).

Though Fr. II eTregs are generally considered stable, activation-associated pathways enriched in eTregs may modulate their stability and function. For example, OX40 signaling can antagonize TGF-β-driven FOXP3 induction and impair regulatory activity under strong costimulatory conditions (201, 202). Similarly, GITR agonism can disrupt Treg-mediated suppression and tolerance and, in tumor models, has been reported to preferentially destabilize intratumoral Treg programs (163, 164, 203). In addition, certain receptors may correlate with instability-prone effector programs: genetic loss of CCR2 signaling has been reported to bias CD4^+^ T cells away from Th1/Th17 cytokine production and toward Treg accumulation *in vivo* and *in vitro* (204). While it remains unclear whether exTregs arise from Th-like Tregs, it has been suggested that, once Tregs adopt Th-like programs, additional stabilization mechanisms may be required to preserve suppressive identity (205).

#### 3.2.4 Context-dependent shifts across physiology and disease

Across physiological states and diseases, Treg proportions, composition, and functionality change dynamically. While reported Treg changes vary substantially, in part because Treg definitions, especially CD25-based gates, can behave differently across inflammatory milieus and treatment exposures (133), a recurring trend is that settings with repeated antigen exposure often show relative enrichment of eTreg states. During aging, multiple studies report a relative contraction of the nTreg pool with enrichment of the eTreg pool, consistent with reduced thymic contribution and cumulative peripheral activation (29, 206, 207). In pregnancy, Tregs expand to support tolerance to fetal and paternal antigens. In mice, repeated pregnancies can further enrich activated/effector Treg states in secondary lymphoid organs and the uterus and improve pregnancy outcomes (208). In autoimmune and chronic inflammatory diseases, most reports show relatively preserved total Treg frequency but reduced eTreg representation or impaired function (148, 209–211), which may be restored by treatment (178).

Tumor-infiltrating Tregs are of substantial interest because they suppress antitumor immunity and represent attractive targets for cancer immunotherapy. Tumors most consistently show dominance of highly activated eTregs among FOXP3^+^ cells (18, 212), a phenotype that may be more pronounced in metastatic tumors, as reported in patients with lung cancer (213). Human tumor-infiltrating Tregs can also display tumor- and neoantigen-reactive TCR repertoires (214). Tumor microenvironments are enriched for chemokines, often arising from oncogenic mutations (162, 215), which recruit chemokine-receptor-expressing eTregs and can further promote highly suppressive effector programs (169, 216, 217) or, in some settings, Th-like Treg states (55). In mouse tumor models, eTreg persistence is further reinforced by niche-specific stabilizing cues and terminal differentiation checkpoints: IL-23R signaling can support the tumor eTreg program (218), MED1 promotes terminal effector differentiation of tumor-supportive Tregs while being dispensable for baseline Treg development, homeostasis, and non-malignant inflammatory responses (219), and BLIMP-1-associated effector modules can sustain intratumoral eTreg suppressive identity and shape responses to checkpoint blockade (220). Metabolic rewiring is also part of the tumor eTreg state: intratumoral Tregs engage a lipid program centered on the SCAP–SREBP axis, supporting fatty-acid synthesis and mevalonate-dependent processes that reinforce suppressive identity (221). In human breast cancer and head-and-neck squamous-cell carcinoma, tumor Tregs also show enrichment of an SREBP-associated transcriptional signature (221).

## 4 Where they go: therapeutic application of different subsets of human Treg cells

### 4.1 Treg ACT: nTregs are the major starting population, and engineering is an emerging strategy

As discussed above, a recurring practical limitation is that highly pure Treg populations are difficult to isolate at scale. Most GMP-compatible *ex vivo* expansion procedures incorporate IL-2 plus rapamycin to suppress Tconv outgrowth while permitting Treg expansion, although a trade-off has been reported in which rapamycin can also reduce Treg proliferation (222–224). In line with this, while early trials mainly used bulk polyclonal (CD4^+^CD127^low/-^CD25^+^) Treg adoptive cell therapy (ACT) and have demonstrated feasible manufacturing and safe administration (132, 225–232), there has been an increasing number of ACT efforts prioritizing products enriched for CD45RA^+^ nTregs, because this compartment is comparatively stable, expands robustly, and is less confounded by activated non-Tregs (166, 233, 234) (**Table 4**). For peripheral blood–derived products, one practical manufacturing improvement is to deplete eTregs prior to expansion, which can improve downstream purity and consistency (166). Umbilical cord blood (UCB) is also an attractive source because it is enriched for naïve-like cells with broad TCR diversity and tends to have fewer activated CD25^+^ contaminants, and early clinical studies have demonstrated feasibility and safety of UCB-derived Treg infusion in transplantation settings (235, 236, 251–253). In addition, pediatric thymic tissue (e.g., discarded thymus from infant cardiac surgery) has been explored as a potentially abundant source of thymus-derived Tregs (223, 254).

**Table 4:** Clinical trials of Treg-directed therapies.

Therapeutic platform	Product name	Modality	Trial (NCT number; acronym)	Indication	Phase	n	Status	Reference
Polyclonal Treg ACT	—	Autologous polyclonal Treg	NCT02129881 (ONETreg1)	Living-donor kidney transplantation, immunosuppression minimisation	1/2	15	Completed	(226)
	—	Autologous naive-enriched nTreg	NCT02371434 (ONEnTreg13)	Living-donor kidney transplantation, immunosuppression minimisation	1/2	17	Completed	(234)
	—	Autologous polyclonal Treg	NCT02145325 (TRACT)	Living-donor kidney transplantation	1	10	Completed	(227)
	—	Autologous polyclonal Treg	NCT02166177 (ThRIL)	Liver transplantation, immunosuppression reduction	1/2	9	Completed	(232)
	—	Autologous polyclonal Treg	NCT02711826 (TASK)	Kidney transplant recipients with subclinical inflammation on surveillance biopsy	1/2	32	Completed	*ClinicalTrials.gov record*
	—	Autologous polyclonal Treg + everolimus	NCT03284242	Kidney transplantation	Early 1	9	Completed	*ClinicalTrials.gov record*
	—	Autologous polyclonal Treg + tocilizumab	NCT03867617	Kidney transplantation	1/2	12	Active, not recruiting	*ClinicalTrials.gov record*
	—	Autologous polyclonal Treg	NCT06777719 (Eight-Treg)	Kidney transplantation	1	9	Recruiting	*ClinicalTrials.gov record*
	PolyTregs	Autologous polyclonal Treg with islet graft	NCT03444064	Type 1 diabetes, islet allotransplantation	1	11	Completed	*ClinicalTrials.gov record;* (132)
	—	Autologous polyclonal Treg with islet graft	NCT05973734	Islet transplantation in type 1 diabetes	1	24	Enrolling by invitation	*ClinicalTrials.gov record*
	PolyTregs	Autologous polyclonal Treg	NCT01210664 (Treg)	Recent-onset type 1 diabetes	1	16	Completed	(132)
	PolyTregs	Autologous polyclonal Treg + low-dose IL-2	NCT02772679 (TILT)	Type 1 diabetes	1	16	Completed	*ClinicalTrials.gov record*
	—	Autologous polyclonal Treg	NCT02428309	Cutaneous lupus erythematosus	1	1	Terminated	*ClinicalTrials.gov record*
	TR004	Autologous polyclonal Treg	NCT03185000 (TRIBUTE)	Crohn disease	1	4	Status unknown	*ClinicalTrials.gov record*
	—	Autologous polyclonal Treg	NCT04691232	Ulcerative colitis	1	11	Completed	(229, 228)
	—	Allogeneic cord-blood Treg	NCT02932826	Type 1 diabetes	1/2	40	Status unknown	*ClinicalTrials.gov record*
	—	Allogeneic cord-blood Treg + liraglutide	NCT03011021	Type 1 diabetes	1/2	40	Status unknown	*ClinicalTrials.gov record*
	—	Autologous polyclonal Treg +/- rituximab	NCT06688331 (PreTreg)	Presymptomatic (stage 1) type 1 diabetes in children and adolescents	2	150	Recruiting	*ClinicalTrials.gov record;* (230)
	—	Autologous polyclonal Treg	NCT06708780	Type 1 diabetes	1	20	Recruiting	*ClinicalTrials.gov record*
	—	Autologous polyclonal Treg	NCT05566977	Lupus nephritis and type 1 diabetes	1	20	Status unknown	*ClinicalTrials.gov record*
	—	Autologous polyclonal Treg	NCT02704338 (Treg)	Autoimmune diseases (basket design)	1/2	30	Status unknown	*ClinicalTrials.gov record*
	—	Autologous polyclonal Treg + low-dose IL-2	NCT03241784	Amyotrophic lateral sclerosis	1	3	Completed	*ClinicalTrials.gov record*
	—	Autologous polyclonal Treg + low-dose IL-2	NCT04055623 (Tregs in ALS)	Amyotrophic lateral sclerosis	2	12	Active, not recruiting	*ClinicalTrials.gov record*
	CK0803	Allogeneic cord-blood Treg	NCT05695521 (REGALS)	Amyotrophic lateral sclerosis	1	66	Active, not recruiting	*ClinicalTrials.gov record*
	—	Autologous polyclonal Treg	NCT06671236	Amyotrophic lateral sclerosis	1	12	Recruiting	*ClinicalTrials.gov record*
	GB301	Autologous polyclonal Treg	NCT03865017	Alzheimer disease	1/2	20	Status unknown	*ClinicalTrials.gov record*
	VT301	Autologous polyclonal Treg	NCT05016427	Alzheimer disease	1	12	Status unknown	*ClinicalTrials.gov record*
	CK0802	Allogeneic cord-blood Treg	NCT04468971 (RESOLVE)	COVID-19-associated ARDS	1	45	Completed	*ClinicalTrials.gov record*
Treg ACT: HSCT and graft-versus-host disease	—	Allogeneic cord-blood Treg	NCT00602693	Haematological malignancy, GvHD prophylaxis after umbilical cord blood transplant	1	41	Completed	(235, 236)
	—	Donor-derived expanded Treg	NCT01795573	GvHD prophylaxis after allogeneic HSCT	1	38	Completed	*ClinicalTrials.gov record*
	—	Donor-derived Treg	NCT01911039	Steroid-dependent or steroid-refractory chronic GvHD	1	14	Completed	*ClinicalTrials.gov record*
	—	Donor Treg-enriched DLI + low-dose IL-2	NCT01937468	Steroid-refractory chronic GvHD	1	25	Active, not recruiting	(231)
	—	Donor-derived Treg DLI, repeated	NCT02749084 (TREG2015001)	Severe refractory chronic GvHD	1/2	18	Completed	*ClinicalTrials.gov record*
	—	Donor Treg-enriched infusion	NCT03683498 (GVHD-TReG)	Steroid-refractory chronic GvHD	1	16	Completed	*ClinicalTrials.gov record*
	—	Donor-derived Treg	NCT06920199	Refractory chronic GvHD	Early 1	18	Recruiting	*ClinicalTrials.gov record*
	EVE-Treg	Donor-derived expanded Treg + low-dose IL-2	NCT06991361 (EVENT)	Chronic GvHD	1	21	Recruiting	*ClinicalTrials.gov record*
	CK0802	Allogeneic cord-blood Treg	NCT07576699	Steroid-refractory GvHD	1/2	12	Not yet recruiting	*ClinicalTrials.gov record*
	—	Treg/Tcon co-infusion with a CD34-selected graft	NCT01660607	Haematological malignancy, HLA-mismatched/haploidentical HSCT	1/2	68	Completed	*ClinicalTrials.gov record*
	—	Purified donor Treg with the graft	NCT05088356	Advanced haematological malignancy, reduced-intensity allogeneic HCT	1	66	Active, not recruiting	*ClinicalTrials.gov record*
	Orca-T (now called Tregzi)	Engineered allograft: CD34^+^ HSPC + sorted Treg + Tcon	NCT04013685	AML, ALL, MDS and related malignancies undergoing myeloablative allogeneic HSCT	1	155	Active, not recruiting	*ClinicalTrials.gov record;* (237)
	Orca-T (now called Tregzi)	Engineered allograft: CD34^+^ HSPC + sorted Treg + Tcon	NCT05316701 (Precision-T)	AML, ALL and MDS, myeloablative HLA-matched allogeneic HSCT	3	187	Active, not recruiting	(238)
	Orca-Q	Engineered allograft for mismatched donors	NCT03802695	Haematological malignancy, allogeneic HSCT from mismatched or haploidentical donors	1	300	Recruiting	*ClinicalTrials.gov record*
Antigen-specific Treg ACT (alloantigen-reactive)	darTregs	Alloantigen-reactive autologous Treg	NCT02474199 (ARTEMIS)	Liver transplant recipients undergoing calcineurin-inhibitor withdrawal	1/2	15	Completed	(239)
	darTregs	Alloantigen-reactive autologous Treg	NCT02244801 (DART)	Living-donor kidney transplantation	1	6	Completed	(240, 226)
TCR-Treg	ABA-101	TCR-Treg (HLA-restricted peptide)	NCT06566261	Progressive multiple sclerosis	1	12	Active, not recruiting	*ClinicalTrials.gov record*
CAR-Treg	TX200-TR101	HLA-A*02-CAR-Treg	NCT04817774 (STEADFAST)	HLA-A*02-mismatched living-donor kidney transplantation	1/2	26	Completed	*ClinicalTrials.gov record*
	QEL-001	HLA-A2-CAR-Treg, multi-module	NCT05234190 (LIBERATE)	HLA-A2-mismatched liver transplantation with immunosuppression withdrawal	1/2	33	Active, not recruiting	*ClinicalTrials.gov record*
	SBT777101	Cit-P-CAR-Treg	NCT06201416 (Regulate-RA)	Refractory rheumatoid arthritis	1	48	Recruiting	*Kohler* et al.*, 2025 (ACR Convergence abstract LB23); ClinicalTrials.gov record*
	SBT777101	Cit-P-CAR-Treg	NCT06361836 (Regulate-HS)	Hidradenitis suppurativa	1	24	Active, not recruiting	*ClinicalTrials.gov record*
	—	CD6-CAR-Treg, allogeneic	NCT05993611	Chronic GvHD after allogeneic HSCT	1	27	Suspended	*ClinicalTrials.gov record*
	—	CD6-CAR-Treg, autologous	NCT07395050	Stage 3 type 1 diabetes	Early 1	6	Not yet recruiting	*ClinicalTrials.gov record*
	BEN301	CAR-Treg, target not disclosed (CD4^+^CAR^+^FOXP3^+^)	NCT07502859	Systemic sclerosis, rheumatoid arthritis, Sjogren disease and idiopathic inflammatory myopathy	Early 1	24	Not yet recruiting	*ClinicalTrials.gov record*
	QEL-005	CD19-CAR-Treg	NCT07473154 (CHILL)	Diffuse cutaneous systemic sclerosis; difficult-to-treat rheumatoid arthritis	1/2	16	Recruiting	*ClinicalTrials.gov record*
	—	CD19-CAR-Treg, allogeneic thymus-derived	NCT05114837	Lymphoid malignancy (GvHD/toxicity control)	1/2	0	Withdrawn	*ClinicalTrials.gov record*
FOXP3-engineered regulatory-like cells (engTregs)	CD4-LVFOXP3	FOXP3-transduced autologous CD4^+^ T cell	NCT05241444	IPEX syndrome (FOXP3 deficiency)	1	30	Recruiting	*ClinicalTrials.gov record*
	FOXP3-T4	FOXP3-transduced autologous CD4^+^ T cell +/- low-dose IL-2	NCT07697118 (THERIPEX)	IPEX syndrome	1/2	5	Not yet recruiting	*ClinicalTrials.gov record*
	GNTI-122	engTreg with islet-antigen specificity (HLA-DRB1*04:01)	NCT06919354 (POLARIS)	Recently diagnosed type 1 diabetes	1	16	Recruiting	*ClinicalTrials.gov record*
*In vivo* Treg expansion by low-dose IL-2	Recombinant IL-2	Low-dose IL-2	NCT01988506 (TRANSREG)	14 autoimmune and auto-inflammatory diseases (basket)	2	81	Completed	*ClinicalTrials.gov record*
	Aldesleukin	Low-dose IL-2	NCT01353833 (DF-IL2)	Type 1 diabetes	1/2	25	Completed	*ClinicalTrials.gov record*
	Recombinant IL-2	Ultra-low-dose IL-2	NCT01862120 (DFIL2-Child)	Recent-onset type 1 diabetes in children	2	24	Completed	*ClinicalTrials.gov record*
	Aldesleukin	Low-dose IL-2	NCT02265809 (DILfrequency)	Type 1 diabetes	1/2	41	Completed	*ClinicalTrials.gov record*
	Aldesleukin (Proleukin)	Low-dose IL-2	NCT03312335 (Charact-IL-2)	Systemic lupus erythematosus	2	16	Completed	*ClinicalTrials.gov record*
	Recombinant human IL-2	Low-dose IL-2	NCT02465580	Active systemic lupus erythematosus	2	60	Status unknown	(241)*; also registered as NCT02932137*
	Recombinant IL-2	Low-dose IL-2	NCT02084238	Systemic lupus erythematosus	Not phased	40	Completed	*ClinicalTrials.gov record*
	Recombinant IL-2	Low-dose IL-2	NCT02424396 (MS-IL2)	Relapsing-remitting multiple sclerosis	2	30	Completed	*ClinicalTrials.gov record*
	Recombinant IL-2	Low-dose IL-2 + riluzole	NCT03039673 (MIROCALS)	Amyotrophic lateral sclerosis	2	304	Completed	(242, 243)
	Recombinant IL-2	Low-dose IL-2	NCT02059759 (IMODALS)	Amyotrophic lateral sclerosis	2	36	Completed	(243)
	ILT-101	Ciclosporin followed by low-dose IL-2	NCT05153070 (DF-IL2-REP)	Recent-onset type 1 diabetes	2	24	Recruiting	*ClinicalTrials.gov record*
	ILT-101	Low-dose IL-2	NCT03776643 (Rhinil-2)	Birch pollen allergic rhinoconjunctivitis	2	24	Completed	*ClinicalTrials.gov record*
	Recombinant human IL-2	Low-dose IL-2	NCT04023149	Pemphigus	2	180	Status unknown	*ClinicalTrials.gov record*
	Recombinant IL-2	Low-dose IL-2	NCT02949492 (LITE)	Liver transplantation	4	6	Terminated	*ClinicalTrials.gov record;* (244)
	Aldesleukin	Low-dose IL-2	NCT05821153	Alzheimer disease	1	8	Completed	*ClinicalTrials.gov record*
	Recombinant IL-2	Low-dose IL-2	NCT06096090	Alzheimer disease	2	40	Status unknown	*ClinicalTrials.gov record*
*In vivo* Treg expansion by Treg-selective IL-2R agonists	Efavaleukin alfa (AMG 592)	IL-2 mutein Fc fusion	NCT04680637	Active systemic lupus erythematosus	2	168	Terminated	*ClinicalTrials.gov record*
	Efavaleukin alfa	IL-2 mutein Fc fusion	NCT04987307	Ulcerative colitis	2	221	Terminated	*ClinicalTrials.gov record*
	Efavaleukin alfa	IL-2 mutein Fc fusion	NCT03422627	Steroid-refractory chronic GvHD	1/2	32	Terminated	*ClinicalTrials.gov record*
	Rezpegaldesleukin (NKTR-358)	PEGylated IL-2 conjugate	NCT06136741 (REZOLVE-AD)	Moderate-to-severe atopic dermatitis	2	396	Active, not recruiting	(245, 246)
	Rezpegaldesleukin	PEGylated IL-2 conjugate	NCT06340360	Alopecia areata	2	94	Active, not recruiting	*ClinicalTrials.gov record*
	Rezpegaldesleukin	PEGylated IL-2 conjugate	NCT07690371 (ZENITH AD-1)	Moderate-to-severe atopic dermatitis	3	510	Recruiting	*ClinicalTrials.gov record*
	Rezpegaldesleukin	PEGylated IL-2 conjugate	NCT07142252	New-onset type 1 diabetes	2	66	Recruiting	*ClinicalTrials.gov record*
	MK-6194 (PT101)	IL-2 mutein Fc fusion	NCT06161116	Systemic lupus erythematosus	2	149	Terminated	*ClinicalTrials.gov record*
	SAR444336	Treg-selective IL-2R agonist	NCT07156175	Microscopic colitis	2	54	Recruiting	*ClinicalTrials.gov record*
Tolerogenic vaccination and tolerogenic dendritic cells	—	Autologous tolerogenic dendritic cell	NCT02252055 (ONEatDC)	Living-donor kidney transplantation	1/2	11	Completed	(226)
	—	Autologous tolerogenic dendritic cell	NCT06852625 (ATDC-PICI)	High-immunological-risk kidney transplantation	1	30	Not yet recruiting	*ClinicalTrials.gov record*
	—	Vitamin D3-conditioned, peptide-loaded tolerogenic DC	NCT02618902 (MS-tolDC)	Multiple sclerosis	1	9	Completed	*ClinicalTrials.gov record*
	—	Antigen-loaded tolerogenic dendritic cell	NCT02283671	Multiple sclerosis and neuromyelitis optica	1	20	Completed	*ClinicalTrials.gov record*
	PIpepTolDC	Proinsulin-peptide-loaded tolerogenic DC	NCT04590872	Type 1 diabetes	1	6	Active, not recruiting	*ClinicalTrials.gov record*
	—	Autologous tolerogenic dendritic cell	NCT05207995	Type 1 diabetes	1/2	32	Completed	*ClinicalTrials.gov record*
	—	Intralesional tolerogenic dendritic cell	NCT02622763 (TolDecCDintra)	Crohn disease (perianal fistulising)	1	3	Terminated	*ClinicalTrials.gov record*
Tumour-Treg depletion: anti-CCR4	Mogamulizumab (KW-0761)	Defucosylated anti-CCR4 mAb	NCT01728805 (MAVORIC)	Relapsed/refractory cutaneous T-cell lymphoma (mycosis fungoides, Sezary syndrome)	3	372	Completed	(247, 248)
	Mogamulizumab (KW-0761)	Defucosylated anti-CCR4 mAb	NCT02281409	Advanced solid tumours	1/2	17	Completed	(249)
	Mogamulizumab + nivolumab	Anti-CCR4 mAb + anti-PD-1	NCT02476123	Advanced solid tumours	1	118	Completed	*ClinicalTrials.gov record*
Tumour-Treg depletion: anti-CCR8	Denikitug (GS-1811)	Anti-CCR8 mAb +/- anti-PD-1	NCT05007782	Advanced solid tumours	1	416	Recruiting	*ClinicalTrials.gov record*
	Tagmokitug (CHS-114)	Anti-CCR8 mAb +/- anti-PD-1	NCT05635643	Advanced solid tumours, including head and neck squamous cell carcinoma	1	87	Active, not recruiting	*ClinicalTrials.gov record*
	BMS-986340	Anti-CCR8 mAb	NCT04895709	Cervical, gastric/gastro-oesophageal, colorectal and other advanced solid tumours	1/2	1109	Recruiting	*ClinicalTrials.gov record*
	S-531011	Anti-CCR8 agent	NCT05101070 (aCCeleR8-001)	Advanced solid tumours	1/2	282	Recruiting	*ClinicalTrials.gov record*
	LM-108	Anti-CCR8 mAb + anti-PD-1	NCT05518045	Advanced solid tumours	1/2	392	Active, not recruiting	*ClinicalTrials.gov record*
	BAY3375968	Anti-CCR8 mAb	NCT05537740	Advanced solid tumours	1	129	Active, not recruiting	*ClinicalTrials.gov record*
	DT-7012	Anti-CCR8 agent	NCT06819735 (DOMISOL)	Advanced solid tumours	1/2	125	Recruiting	*ClinicalTrials.gov record*
Tumour-Treg depletion: other targets	Isatuximab + cemiplimab	Anti-CD38 mAb + anti-PD-1	NCT04763616 (ICING)	Relapsed/refractory natural killer/T-cell lymphoma	2	37	Completed	*ClinicalTrials.gov record*
Tumour-Treg depletion: anti-CD25	Vopikitug (RO7296682)	IL-2-sparing anti-CD25 mAb	NCT04158583	Advanced solid tumours	1	76	Terminated	*ClinicalTrials.gov record*
	Camidanlumab tesirine	Anti-CD25 antibody-drug conjugate	NCT04052997	Relapsed/refractory classic Hodgkin lymphoma	2	117	Completed	(250)
Tumour-Treg depletion: graft engineering	—	CD25-high Treg-depleted DLI + ipilimumab	NCT03912064	Relapsed AML/MDS after allogeneic HSCT	1	25	Active, not recruiting	*ClinicalTrials.gov record*
	—	Treg-depleted DLI	NCT03236129 (DLI-Boost)	Haematological malignancy after allogeneic HSCT	3	52	Status unknown	*ClinicalTrials.gov record*

Data accessed on 2 September 2026.

A major limitation of polyclonal nTreg products is that only a minority of infused cells carry the “right” specificity to the relevant antigen, and despite the ability of Tregs to mediate bystander suppression (8), insufficient TCR engagement can dilute potency. Antigen specificity can be selected rather than engineered: recipient Tregs stimulated *ex vivo* with donor B cells or donor alloantigen yield donor-alloantigen-reactive Tregs (darTregs), which suppress the specific alloresponse far more potently than polyclonal Tregs while retaining a diverse TCR repertoire and a stable Treg phenotype and are currently evaluated in clinical trials (239, 240) (**Table 4**). Manufacturing yield, rather than safety, is therefore the limiting factor for this platform. These motivate antigen-targeted Treg engineering, including transgenic TCR-expressing Tregs (TCR-Tregs) and chimeric antigen receptor-expressing Tregs (CAR-Tregs), which aim to enhance antigen-dependent trafficking, retention, and local activation (255–257) (**Figure 3**). TCR-Tregs recognize cognate peptide–HLA complexes, typically HLA class II complexes on professional antigen-presenting cells, thereby enabling regulation at sites of antigen presentation and pathogenic CD4^+^ T-cell priming; however, this approach requires compatibility with the restricting HLA allele (255–257). In contrast, CAR-Tregs recognize intact, accessible surface antigens independently of peptide processing and HLA restriction, allowing targeting of antigen-expressing graft, tissue, stromal, or immune cells (255–257). In both approaches, antigen engagement localizes and activates the Treg product, which can then suppress co-localized immune responses through canonical Treg mechanisms and bystander suppression beyond the directly recognized antigen (8, 255, 257). Beyond CAR and TCR, engineering strategies also involve modulating gene expressions to improve stability, trafficking, and persistence, including enforced expression of *FOXP3*, sometimes together with *HELIOS*, which has been explored to improve the stability, efficacy, and safety of human CAR-Treg products (258, 259).

**Figure 3:**
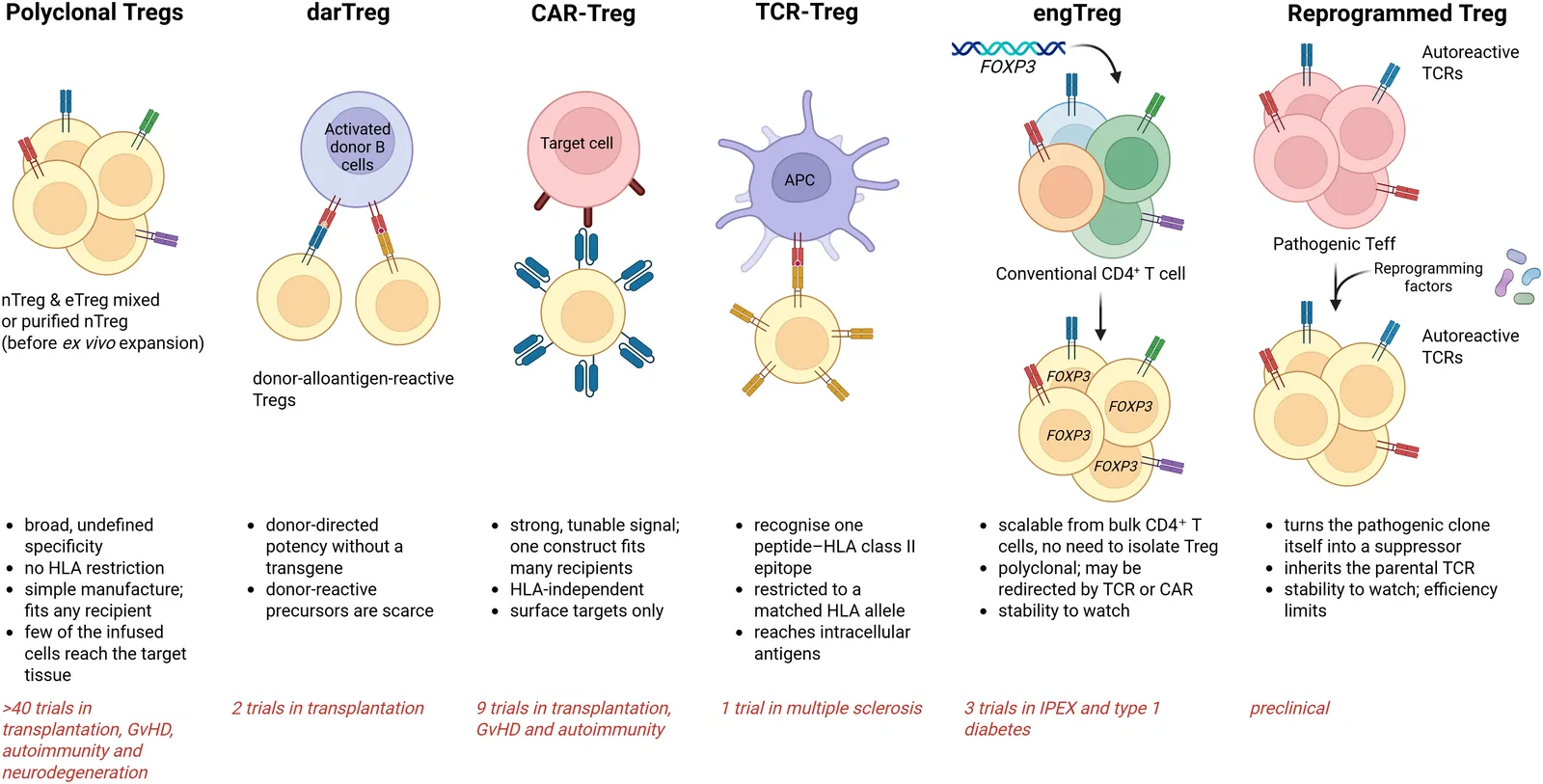
Major Treg ACT platforms. The six platforms are shown in order of increasing engineering, from selected to engineered specificity and finally engineered identity. Polyclonal Treg products are initiated with mixed nTregs and eTregs, or of purified nTregs, and then expanded *ex vivo*; they carry broad, undefined specificity and require no HLA restriction, which keeps manufacturing simple and applicable to any recipient, but only a minority of the infused cells reach the target tissue. darTregs are selected rather than engineered: recipient Tregs are stimulated *ex vivo* with activated donor B cells or donor alloantigen and the donor-reactive cells are expanded, giving donor-directed potency without a transgene, at the cost of depending on scarce donor-reactive precursors. CAR-Tregs bind an intact cell-surface antigen independently of HLA, which provides a strong, tunable signal from a single construct that fits many recipients but restricts targeting to accessible surface antigens. TCR-Tregs recognize one processed peptide presented by HLA class II, which gives access to intracellular antigens but restricts use to recipients carrying the matched allele. engTregs are generated by installing FOXP3 in bulk conventional CD4^+^ T cells, which are scalable and do not require isolation of rare endogenous Tregs, and can be redirected by a transgenic TCR or CAR; the durability of induced FOXP3 expression remains the main uncertainty. Reprogrammed Tregs are pathogenic effector T cells converted into FOXP3^+^ suppressors that inherit the parental autoreactive TCR, thereby turning the pathogenic clone itself into a suppressor, with reversion risk and conversion efficiency as the principal limitations. The number of registered clinical trials shown for each platform (italics) is that of **Table 4**, verified against ClinicalTrials.gov in September 2026. APC, antigen-presenting cell; CAR, chimeric antigen receptor; darTreg, donor-alloantigen-reactive Treg; engTreg, FOXP3-engineered regulatory-like cell; eTreg, effector Treg; GvHD, graft-versus-host disease; IPEX, immune dysregulation, polyendocrinopathy, enteropathy, X-linked syndrome; nTreg, naïve Treg; TCR, T cell receptor; Teff, effector T cell.

Treg ACT has now reached its first regulatory approval, from the least engineered end of the spectrum: TREGZI (Orca Bio) — an 8/8 HLA-matched graft given as separate infusions of purified CD34^+^ cells, a defined dose of freshly isolated, unexpanded CD4^+^CD25^+^CD127^−/low^ donor Tregs, and conventional T cells — was approved by the FDA on 30 June 2026 after improving cGVHD-free survival in the phase 3 Precision-T study (NCT05316701, **Table 4**) (237, 238), making it the first approved cell therapy to include a defined Treg dose. It sidesteps Treg heterogeneity rather than resolving it, leaving nTreg/eTreg composition undefined while showing that reproducible dosing and manufacturing simplicity can be decisive for translation.

### 4.2 Treg ACT: harnessing specialized eTreg programs and non-Treg cells

Although many current ACT manufacturing workflows start from CD45RA^+^ nTregs, the expanded product is phenotypically activated and eTreg-like at the time of infusion (29). However, these polyclonal eTregs are not necessarily skewed to the targeted tissue and immune pathology. A precision strategy for Treg ACT is therefore to harness the small population of pre-existing specialized eTregs that are already wired for context-matched suppression. Early conceptual studies purified, sometimes expanded, and reinfused eTregs such as CXCR3^+^ Th1-like Tregs for Th1-dominant pathology (260), Tfr-like populations for antibody-driven autoimmunity (261), and CD103-expressing Tregs for gut autoimmune conditions (28, 187). Though conceptually compelling, direct use of pre-existing eTregs presents practical challenges because these populations can be heterogeneous, less abundant, and potentially more susceptible to activation-induced functional drift than products manufactured from nTreg-enriched inputs. In parallel, a rapidly developing field aims to generate stable Th-like FOXP3^+^ eTregs from conventional effector T cells, including pathogenic effector T cells, using reinforced reprogramming protocols as discussed above, thereby expanding the feasible supply of context-matched eTreg products for ACT (116–118) (**Figure 3**). These strategies could expand the supply of antigen- and context-matched regulatory products, but they require stringent evaluation of lineage stability, epigenetic identity, trafficking, and safety before clinical translation.

On the other hand, FOXP3-engineered regulatory-like cells (engTregs) provide an alternative to isolating and expanding rare endogenous Tregs. In this approach, bulk conventional CD4^+^ T cells are engineered to sustain *FOXP3* expression, generating cells with Treg-associated markers and suppressive activity (**Figure 3**). The approach has evolved to include targeted editing at the endogenous *FOXP3* locus and promoter editing to provide durable expression, and edited cells showed suppressive function in *in vitro* assays and xenogeneic graft-versus-host disease models (262). Additional engineering, including a rapamycin-responsive chemically inducible signaling complex (CISC), can provide controllable IL-2-like signaling to support selective expansion, persistence, and efficacy *in vivo* (263). engTregs can also incorporate antigen-specific TCRs or CARs; for example, islet antigen-specific engTregs reduced insulitis and prevented diabetes in a mouse model, whereas CD19-directed allogeneic engTregs are being explored for B cell-mediated autoimmunity (264, 265). Ongoing clinical studies in IPEX syndrome (NCT05241444) and type 1 diabetes (NCT06919354) will help define the safety, stability, and therapeutic potential of these products (**Table 4**), while their relationship to the broader transcriptional and epigenetic program of naturally derived Tregs remains an important area of investigation (4–7).

### 4.3 Pharmacological modulation of Tregs: IL-2-based therapies, tolerogenic vaccines, cancer immunotherapy, and beyond

Low-dose IL-2 was the first widely tested pharmacological approach, leveraging their constitutively high CD25 (IL-2Rα), to preferentially expand Tregs *in vivo* and restore immune regulation (266, 267). The method has since matured conceptually and clinically (241–244, 267, 268), with recent extensions into non-classical inflammatory settings such as acute coronary syndromes (269). To improve pharmacology and selectivity, IL-2/anti-IL-2 antibody complexes have been developed to enhance the *in vivo* half-life of IL-2, and multiple CD25-biased IL-2 muteins have been developed to enhance Treg-targeting (268, 270). Another example of engineered IL-2 is Rezpegaldesleukin (REZPEG; NKTR-358), which is a PEG-modified recombinant human IL-2 molecule, rather than a conventional low-dose formulation of unmodified IL-2; PEGylation prolongs systemic exposure and is intended to preferentially stimulate CD25^high^ Tregs (246, 271, 272). Recent phase 2b data in adults with moderate-to-severe atopic dermatitis suggest that REZPEG improves clinical disease activity and is associated with expansion of circulating Tregs (245). Because both nTregs and eTregs express CD25, whereas eTregs often express it at higher levels, IL-2-based interventions may preferentially expand activated or tissue-homing eTreg subsets. Consistent with this possibility, a recent high-resolution profiling of patients with SLE showed that low-dose IL-2 therapy preferentially expanded activated and homing-defined eTreg subsets (178).

In addition to systemic cytokine-based Treg expansion, tolerogenic vaccines aim to restore antigen-specific immune tolerance by delivering disease-relevant antigens in a non-inflammatory or actively tolerogenic context, thereby engaging tolerogenic antigen-presenting cells and biasing T cell fate toward regulatory rather than effector programs (273). Proposed mechanisms include induction of tolerogenic dendritic cells, deletion or anergy of pathogenic lymphocytes, production of regulatory cytokines such as IL-10 and TGF-β, and expansion of antigen-specific FOXP3^+^ Tregs and other regulatory populations (273, 274). Platforms under investigation include soluble or modified antigens, peptide-coupled cells, tolerogenic dendritic cells, nanoparticles or microparticles co-delivering antigen and tolerogenic cues, and non-inflammatory mRNA-based approaches. Clinical experience is most extensive for tolerogenic dendritic cells, which have completed early-phase trials in type 1 diabetes, rheumatoid arthritis, multiple sclerosis and kidney transplantation (**Table 4**), whereas other approaches remain preclinical. For example, non-inflammatory mRNA vaccination encoding myelin antigens induced antigen-specific Tregs and protected against EAE in mice (274). Similarly, nanoparticles co-delivering MOG peptide and an aryl hydrocarbon receptor ligand promoted tolerogenic dendritic cells, induced antigen-specific FOXP3^+^ Tregs, and suppressed EAE (275). A distinct GM-CSF–neuroantigen fusion vaccine expanded myelin-reactive CD4^+^CD25^+^FOXP3^+^ Tregs and inhibited EAE, illustrating how antigen presentation can be tuned to favor regulatory rather than effector responses (276).

In addition to boosting Tregs to control pathogenic immunity, attenuating their activity in tumor environments is a compelling approach to enhance anti-tumor immunity (217). This goal has gained further importance because checkpoint blockade, especially PD-1-targeted therapy, can in some settings expand or potentiate PD-1^+^ Tregs and has been associated with hyperprogressive disease or impaired protective immunity (154, 277, 278). Because non-selective Treg depletion risks breaking systemic immune homeostasis (279), there is strong interest in strategies that preferentially target tumor eTregs while sparing other Tregs. Examples include depletion approaches targeting tumor eTreg-enriched surface programs, such as CCR4 (41, 247–249), CCR8 (169, 280), CD74 (281), and CD38 (177, 190), as well as CD25 targeted by an antibody-drug conjugate (250) (**Table 4**). Finally, the unique biology of the tumor eTreg niche may create therapeutic vulnerabilities: a recent clinical-translational profiling study suggests that PARP inhibition can remodel the tumor immune ecosystem in a way that selectively targets eTregs, particularly in homologous-recombination–deficient ovarian cancer (282).

## 5 Summary and discussion

Treg heterogeneity has been recognized since the earliest descriptions of Tregs, and it has drawn increasing attention as the field has gained the tools to resolve Tregs into multiple subsets. In this review, we addressed “who they are, where they come from, and where they go” by clarifying the defining features of major Treg states, summarizing the core differentiation and trafficking routes that generate these states, and discussing how this biology informs therapeutic opportunities, with emphasis on the nTreg–eTreg axis.

nTregs and eTregs play complementary roles. nTregs function as a comparatively stable and renewable reservoir that sustains long-term tolerance and can expand into downstream states when appropriately stimulated, whereas eTregs represent the frontline regulatory compartment that concentrates suppressive machinery and homing programs needed for active control at priming sites and in tissues, including in inflamed organs and tumors. The same plasticity that enables eTregs to specialize also potentially introduces risks, and a key outstanding question is whether these specialized states represent stable functional endpoints, transient adaptations to local cues, or, in particular inflammatory settings, intermediates that may precede functional impairment or loss of regulatory identity.

The origins and stability of human pTregs, iTregs, and exTregs also remain important unresolved areas. Mouse studies provide strong mechanistic evidence that tolerogenic antigen presentation, cytokines, metabolites, and costimulatory conditions can generate or stabilize regulatory programs, but the extent to which these pathways operate in human disease remains less clear. Recent reprogramming studies demonstrate that effector/memory T cells can be converted into functionally suppressive FOXP3^+^ cells when FOXP3 induction is coupled with conditions that support broader epigenetic remodeling. These findings raise the possibility of repurposing pathogenic, tissue-reactive, or tumor-reactive repertoires toward regulation, but also emphasize the need to establish durable identity and safety under inflammatory conditions. Likewise, exTreg formation is well supported by murine fate-mapping models, whereas its frequency, origin, and pathological relevance in humans remain difficult to define prospectively.

It is also notable that Treg heterogeneity is best understood as the result of overlapping developmental, activation, migratory, and tissue-adaptation programs rather than as a set of fixed and fully separable cell types. The nTreg–eTreg framework provides a practical basis for interpreting human Treg biology, but it does not itself define developmental origin or predict function in every setting. A major methodological challenge is that human Treg identity cannot be assigned by any single marker. Activated conventional T cells can transiently express FOXP3, CD25, CTLA4, and reduced CD127, complicating interpretation of activated CD45RA^−^ populations. Although combinations of CD45RA, CD25, and CD127 can distinguish relatively pure nTreg and eTreg populations in blood, this framework is less definitive in inflamed tissues, tumors, and manipulated cell products. Similarly, no individual marker reliably discriminates human tTregs from pTregs. Future studies should therefore combine phenotypic definition with functional assays, TCR repertoire analysis, transcriptional and chromatin profiling, and DNA-methylation measurements. Such integrated analyses will be particularly important for determining whether Tregs identified in blood correspond to the tissue-adapted populations that directly regulate local inflammation, tissue repair, graft responses, or antitumor immunity.

Treg heterogeneity, especially the distinction between nTregs and eTregs, matters because many ACT interventions are subset biased. nTreg-enriched starting populations currently offer practical manufacturing advantages, including relative purity, proliferative capacity, and lower contamination by activated conventional T cells. Nevertheless, expansion typically produces an activated eTreg-like product, and their diverse TCR repertoire may make inhibition rely largely on bystander mechanisms. This shortcoming can be complemented by TCR-Treg and CAR-Treg strategies, which may improve localization and activation at sites of relevant antigen recognition. In addition, specialized eTregs, reprogrammed Tregs, and FOXP3-engineered regulatory-like cells may provide more context-matched or antigen-specific suppression, but their potential benefits must be balanced against challenges in product consistency, persistence, trafficking, and functional stability.

Pharmacological approaches likewise require a more subset- and context-resolved understanding. Low-dose IL-2 and engineered IL-2 modalities can expand endogenous Tregs, but their effects likely depend on receptor bias, exposure, dose, inflammatory state, and access to tissue compartments. The preferential expansion of activated and homing-defined eTreg subsets observed following low-dose IL-2 in SLE illustrates that these therapies may alter Treg composition rather than simply increase total Treg number. Tolerogenic vaccines and pTreg-inducing approaches provide a complementary route toward antigen-specific immune regulation, but most evidence remains preclinical. Their translation will require solutions to challenges including uncertain target antigens, epitope spreading, disease-stage-dependent inflammation, and demonstration of durable tolerance in heterogeneous human disease. Finally, Treg-directed therapies must accommodate the opposing therapeutic goals in autoimmunity and cancer. In autoimmunity, transplantation, and inflammatory disease, the aim is to expand, induce, or deliver stable Tregs with appropriate antigen specificity and tissue localization. In cancer, the aim is to selectively attenuate highly activated tumor eTregs while preserving systemic immune homeostasis and protective tissue-resident Treg functions. Progress will therefore depend less on broadly increasing or depleting total Tregs than on defining and manipulating the appropriate Treg state in the appropriate anatomical and disease context. Longitudinal, tissue-informed human studies that link Treg phenotype, clonality, functional activity, persistence, and clinical outcome will be essential for translating the expanding understanding of Treg heterogeneity into precise and durable therapies.
